# Local and systemic immune profiles of human pancreatic ductal adenocarcinoma revealed by single-cell mass cytometry

**DOI:** 10.1136/jitc-2022-004638

**Published:** 2022-07-06

**Authors:** Thomas P Brouwer, Natasja L de Vries, Tamim Abdelaal, Ricki T Krog, Zheng Li, Dina Ruano, Arantza Fariña, Boudewijn P F Lelieveldt, Hans Morreau, Bert A Bonsing, Alexander L Vahrmeijer, Frits Koning, Noel F C C de Miranda

**Affiliations:** 1 Department of Pathology, Leiden University Medical Center, Leiden, The Netherlands; 2 Department of Surgery, Leiden University Medical Center, Leiden, The Netherlands; 3 Department of Immunology, Leiden University Medical Centre, Leiden, The Netherlands; 4 Pattern recognition and Bioinformatics, Delft University of Technology, Delft, The Netherlands; 5 Leiden Computational Biology Center, Leiden University Medical Center, Leiden, The Netherlands; 6 Department of Pathology, Cancer Center Amsterdam, Amsterdam, The Netherlands

**Keywords:** tumor microenvironment, gastrointestinal neoplasms, lymphocytes, tumor-infiltrating

## Abstract

**Background:**

Pancreatic ductal adenocarcinoma (PDAC) is a highly lethal malignancy in need of effective (immuno)therapeutic treatment strategies. For the optimal application and development of cancer immunotherapies, a comprehensive understanding of local and systemic immune profiles in patients with PDAC is required. Here, our goal was to decipher the interplay between local and systemic immune profiles in treatment-naïve patients with PDAC.

**Methods:**

The immune composition of PDAC, matched non-malignant pancreatic tissue, regional lymph nodes, spleen, portal vein blood, and peripheral blood samples (collected before and after surgery) from 11 patients with PDAC was assessed by measuring 41 immune cell markers by single-cell mass cytometry. Furthermore, the activation potential of tumor-infiltrating lymphocytes as determined by their ability to produce cytokines was investigated by flow cytometry. In addition, the spatial localization of tumor-infiltrating innate lymphocytes in the tumor microenvironment was confirmed by multispectral immunofluorescence.

**Results:**

We found that CD103^+^CD8^+^ T cells with cytotoxic potential are infrequent in the PDAC immune microenvironment and lack the expression of activation markers and checkpoint blockade molecule programmed cell death protein-1 (PD-1). In contrast, PDAC tissues showed a remarkable increased relative frequency of B cells and regulatory T cells as compared with non-malignant pancreatic tissues. Besides, a previously unappreciated innate lymphocyte cell (ILC) population (CD127^–^CD103^+^CD39^+^CD45RO^+^ ILC1-like) was discovered in PDAC tissues. Strikingly, the increased relative frequency of B cells and regulatory T cells in pancreatic cancer samples was reflected in matched portal vein blood samples but not in peripheral blood, suggesting a regional enrichment of immune cells that infiltrate the PDAC microenvironment. After surgery, decreased frequencies of myeloid dendritic cells were found in peripheral blood.

**Conclusions:**

Our work demonstrates an immunosuppressive landscape in PDAC tissues, generally deprived of cytotoxic T cells and enriched in regulatory T cells and B cells. The antitumor potential of ILC1-like cells in PDAC may be exploited in a therapeutic setting. Importantly, immune profiles detected in blood isolated from the portal vein reflected the immune cell composition of the PDAC microenvironment, suggesting that this anatomical location could be a source of tumor-associated immune cell subsets.

**What is already known on this topic**

- Pancreatic ductal adenocarcinoma (PDAC) is characterized by the lack of naturally occurring antitumor T cell responses due to a low mutational burden and a large stromal compartment including highly immunosuppressive cells.
- The advent of T cell checkpoint blockade has not made an impact in the clinical management of patients with PDAC as PDAC is refractory to this therapy.
- The development of immunotherapeutic strategies for PDAC requires a comprehensive analysis of local and systemic immune profiles in order to identify targetable immune-related features in these cancers.

**What this study adds**

- Our results demonstrate a marked reduction in the frequency of CD103^+^, memory CD8^+^ T cells, accompanied by an increase in the frequency of memory B cells and regulatory T cells in PDAC as compared with non-malignant pancreatic tissues.
- We discovered the presence of an activated, CD103^+^ innate lymphoid cell (ILC)1-like population in PDAC tissues that was infrequent in matched non-malignant, lymph node, and blood samples.
- The immune profiles in PDAC tissue were mimicked in portal vein blood but not peripheral blood samples of the patients.

**How this study might affect research, practice, or policy**

- The findings presented here confirm the unconventional nature of the immune microenvironment in PDAC and encourage further investigation of the functional role of ILC1-like cells and B cells in PDAC, and their possible contribution to immunotherapeutic response.
- The regional enrichment in portal vein blood of immune cells infiltrating the PDAC microenvironment may facilitate a novel source of tumor-associated immune cells that could be exploited in the context of adoptive T cell transfer.

## Background

Pancreatic ductal adenocarcinoma (PDAC) is one of the most lethal cancer types in the industrialized world.^1^ The outlook for patients with PDAC is bleak with a 5-year survival rate of 10%, and without novel therapeutic strategies the survival rate will not improve during the coming years.^2–4^ The development of cancer immunotherapy, using therapeutic antibodies targeting cytotoxic T-lymphocyte-associated protein 4 (CTLA-4) and the programmed cell death protein-1 (PD-1)/programmed death-ligand 1 (PD-L1) axis in particular, have yielded sustained clinical responses in traditionally incurable cancer types including non-small cell lung cancer and melanoma, among others.^5–8^ These immunotherapeutic strategies reinvigorate antitumor T cell responses. PDAC, however, is non-immunogenic and characterized by a lack of naturally occurring immune responses due to a generally low mutational burden as well as a large stromal compartment consisting of few and mostly immunosuppressive immune cells compared with other cancer types,^9^ thereby rendering the cancer insensitive to immunotherapy.^10–12^

Novel omics-technologies can aid in the improvement of patient selection for immunotherapy by giving a more profound insight into the tumorigenic and immunologic mechanisms at play in the tumor microenvironment. With the rise in T cell checkpoint blockade therapies, the T cell landscape has been predominantly looked into. In PDAC, studies have provided insights into the prevalence and distribution of T cells in PDAC tissues and revealed that the T cell landscape is highly complex and varies across patients.^13–16^ Unfortunately, these new insights regarding the immune landscape of PDAC have not impacted clinical responses or survival, with the exception of mismatch repair (MMR)-deficient PDAC tumors.^17–21^

To improve our understanding of additional biological features that could play a role in the lack of response to current immunotherapies and for the development of alternative therapeutic approaches, it is pivotal to determine the immune processes mediating antitumor responses within the local and systemic environment. Here we aimed to decipher the interplay between the local and systemic immune landscapes across PDAC tumors and their matched non-malignant pancreatic tissue, regional lymph nodes, portal vein blood, and peripheral blood obtained before and after surgery from treatment-naïve patients with PDAC. We discovered the presence of an activated, CD103^+^ innate lymphoid cell (ILC)1-like population in PDAC tissues that was infrequent in matched non-malignant, lymph node, and blood samples. Furthermore, we found that PDAC tumors had notably reduced CD103^+^ memory CD8^+^ T cells in comparison to non-malignant pancreatic tissue, while memory B cells and regulatory T cells were significantly increased. The immune profiles in PDAC tissue were mimicked in portal vein blood but not peripheral blood samples of the patients.

## Methods

### Human samples

A total of 11 patients with PDAC, of which none received neoadjuvant chemo(radio)therapy, were recruited at the Leiden University Medical Center through informed consent. Fresh unfixed primary PDAC tissues (N=11) with matched regional lymph nodes adjacent to the common hepatic artery, hepatoduodenal ligament or abdominal aorta (N=19), adjacent non-malignant pancreatic tissue (N=6), spleen (N=2), presurgical and postsurgical peripheral blood samples (N=11 and N=10, respectively, with postsurgical blood withdrawn between 3 and 6 days post operatively), as well as portal vein blood samples (N=9) and 1-year postoperative blood (N=1) were processed for this study (table 1, online supplemental figure 1). Guided by specialized pathologists (AF and HM) macroscopic sectioning was performed to define the tumoural areas for specimen retrieval and subsequent further processing. After sectioning, the chosen tumor samples retrieved were partially snap frozen. The fresh frozen sections were cut and stained with H&E to determine type of tumor, tumor percentage as well as other factors that might play a role in the immune cell composition (table 2).

10.1136/jitc-2022-004638.supp1 Supplementary data

**Table 1:** Different types of samples collected from respective patients

Sample	Tumor	Normal	Lymph node 1	Lymph node 2	Lymph node 3	Spleen	PBMC portal	PBMC presurgery	PBMC postsurgery	PBMC 1 year postsurgery
ISPIC1	+		+	+			+	+	+	+
ISPIC5	+	+	+	+	+		+	+	+	
ISPIC12	+	+				+		+	+	
ISPIC17	+		+	+	+		+	+	+	
ISPIC18	+	+	+	+			+	+	+	
ISPIC20	+	+	+	+			+	+		
ISPIC21	+					+		+	+	
ISPIC22	+	+		+	+		+	+	+	
ISPIC23	+		+		+		+	+	+	
ISPIC24	+		+				+	+	+	
ISPIC26	+	+	+		+		+	+	+	

Lymph node 1, arteria hepatica; lymph node 2, ligamentum hepatoduodenale; lymph node 3, aortacaval.

PBMC, peripheral blood mononuclear cells.

**Table 2:** Pathological characteristics of resected specimens from the respective patients

Sample	Gender	Age (years)	Stage	Location	TNM	Type of specimen	Neoadjuvant (chemo)therapy	History of malignancy	Type of malignancy	Mucine	Pancreatitis in tumor specimen	Dirty necrosis	TLS
ISPIC1	Male	52	IIB	Head	pT1N1M0	PPPD/Whipple	No			+	–	–	+
ISPIC5	Female	49	IIB	Head	pT3N1M0	PPPD/Whipple	No			–	–	+	–
ISPIC12	Male	64	IIB	Tail	pT2N1M0	Lap.distal pancreatectomy	No	Yes	Colon	–	+	–	–
ISPIC17	Male	68	IA	Head	pT1cN0M0	PPPD/Whipple	No			+	+	+	+
ISPIC18	Female	75	IIB	Head	pT1cN2M0	PPPD/Whipple	No			–	–	+	–
ISPIC20	Male	73	IB	Head	pT2N0M0	PPPD/Whipple	No			–	–	–	–
ISPIC21	Female	68	IIB	Tail	pT2N2M0	Open distal pancreatectomy	No			+	–	–	–
ISPIC22	Male	77	IV	Head	pT2N2M1	PPPD/Whipple	No	Yes	Prostate	–	–	–	–
ISPIC23	Male	54	IIB	Head	pT2N2M0	PPPD/Whipple	No			–	–	+	+
ISPIC24	Male	71	IIB	Head	pT2N1M0	PPPD/Whipple	No			–	–	+	–
ISPIC26	Male	54	IIB	Head	pT2N2M0	PPPD/Whipple	No			–	–	+	–

PPPD, pylorus-preserving pancreaticoduodenectomy; TLS, tertiary lymphoid structure; TNM, tumor, node, metastasis.

### Tissue processing

As mentioned in the previous section, a fraction of the tumor samples was snap-frozen, another part was cut into small fragments and digested using 1 mg/mL collagenase D (Roche, Basel, Switzerland) and 50 µg/mL DNAse I (Roche) in IMDM medium (Lonza BioWhittaker, Breda, The Netherlands) supplemented with 2 mM GlutaMAX (Thermo Fisher Scientific, Waltham, Massachusetts, USA), 20% fetal bovine serum (Sigma-Aldrich, Saint Louis, Missouri, USA), 1% penicillin/streptomycin (Thermo Fisher Scientific), 1% Fungizone (Thermo Fisher Scientific), 0.1% ciprofloxacin (provided by the Leiden University Medical Center (LUMC) pharmacy), and 0.1% gentamicin (Sigma-Aldrich). Tissue fragments were incubated for 30 min at 37°C interrupted by three mechanical dissociations on a gentleMACS Dissociator (Miltenyi Biotec, Bergisch Gladback, Germany) in gentleMACS C tubes (Miltenyi Biotec), and subsequently processed through a 70 µm strainer (Miltenyi Biotec). In parallel, the lymph node tissue and spleen samples were processed in the same way. Single cell digests and remaining tumor fragments were cryopreserved for analysis and culturing at later stages.

Blood samples were obtained at their respective time points. Peripheral blood mononuclear cells (PBMC) were isolated from patients’ heparinized venous blood by Ficoll-Amidotrizoate (provided by the LUMC pharmacy) gradient centrifugation.

### Mass cytometry antibody staining and data acquisition

To decipher the immune composition in the different samples from patients with PDAC, mass cytometric analysis was performed with 41 immune cell markers covering immune lineage markers, Fc/complement receptors, differentiation/activation markers, cytokine/chemokine receptors, immunomodulatory molecules, and adhesion/homing molecules (online supplemental figure 1). Briefly, single-cell suspensions were thawed, after which Percoll (GE Healthcare) density-gradient centrifugation was performed to isolate immune cells from PDAC and non-malignant pancreatic tissue. Cells were washed in Maxpar Cell Staining Buffer (CSB, Fluidigm) and counted. Up to 3 million cells of each sample were incubated with 1 mL CSB containing 1 µM Cell-ID intercalator-103Rh (Fluidigm) for 15 min at room temperature (rT) to discriminate dead from live cells. Cells were washed in CSB, incubated with human Fc receptor block (BioLegend) for 10 min at rT, and stained with a cell-surface antibody cocktail for 45 min at rT in a final volume of 100 µL. The antibodies are listed in online supplemental table 1. Purified antibodies were conjugated in-house with heavy metal reporters using the MaxPar X8 Antibody Labeling Kit (Fluidigm) according to the manufacturer’s instructions. All antibodies were titrated to determine the optimal concentration. After three washing steps in CSB, cells were incubated with 1 mL Maxpar Fix and Perm buffer (Fluidigm Sciences) containing 0.125 µM Cell-ID intercalator-Ir (Fluidigm) overnight at 4°C to discriminate singlets from doublets. The next day, cells were washed three times in CSB, and one time in de-ionized water immediately prior to data acquisition. Cells were acquired on a Helios mass cytometer (Fluidigm) at an event rate of <500 events/s in de-ionized water containing 10× diluted EQ Four Element Calibration Beads (Fluidigm). Data were normalized with the normalization passport EQ-P13H2302_ver2 for each experiment.

### Mass cytometry data analysis

Normalized FCS files were analyzed in the FlowJo software V.10.6.1 (Tree Star). Data were checked for quality of staining and gated for live, single, CD45^+^ cells using the 191/193Ir DNA intercalator, CD45, residual, center, width, event length, 103Rh DNA intercalator, and 140Ce bead channels (gating strategy shown in online supplemental figure 1). The final CD45^+^ gate was exported for each sample as FCS file for downstream analysis (online supplemental figure 1). To account for technical variation, a PBMC reference sample was included in every mass cytometry experiment (batch). ComBat was applied to correct for batch effects by aligning the PBMC reference samples and corresponding patient samples across all batches.^22^ CD45^+^ cells were hyberbolic ArcSinh transformed with a cofactor of 5, sample-tagged, and subjected to dimensionality reduction analysis in the Cytosplore software.^23^ All 41 antibodies in the panel showed consistent staining over time and were included in the analysis.

First, CD45^+^ data from all samples (18.2×10^6^ cells) were subjected to a 5-level hierarchical stochastic neighbor embedding (HSNE) analysis^24 25^ with default perplexity and iterations. One portal vein blood sample with an outlying number of CD45^+^ cells (3941; ISPIC5) was excluded from further data analysis. Due to the large number of input cells, we first identified global clusters of naïve CD8 T cells, CD8 memory T cells, naïve CD4 T cells, memory CD4 T cells, γδ T cells, Lin^–^CD7^+^ ILCs, B cells, and myeloid cells (online supplemental figure 2). These clusters were separately analyzed in a data-driven manner up to a maximum number of 0.5×10^6^ cells or landmarks. Clustering of the cells was performed with the Gaussian mean shift algorithm, and clusters that showed high similarity in ArcSinh5-transformed median expression of all markers were merged. Second, CD45^+^ cells from PDAC and non-malignant pancreatic tissue (N=11 and N=6, respectively) were subjected to a t-distributed stochastic neighbor embedding (t-SNE) analysis^26^ with a total of 155 433 cells analyzed with default perplexity (30) and iterations (1000). Clustering of the cells with a sigma value of 20 resulted in the identification of different immune subsets across the major immune lineages of CD4^+^ T cells (N=18), CD8^+^ T cells (N=16), CD25^+^CD127^low^ICOS^+^ CD4^+^ T cells (N=1), γδ T cells (N=1), Lin^–^CD7^+^ ILCs (N=9), B cells (N=7), and myeloid cells (N=16). Absolute cell counts of these immune cell clusters are provided in online supplemental figure 3. Third, CD45^+^ data from all blood samples (10.9×10^6^ cells) were subjected to a 5-level HSNE analysis with default perplexity and iterations. Here, we also first identified global clusters of the major immune lineages, which were separately analyzed in a data-driven manner up to a maximum number of 0.5×10^6^ cells or landmarks. Clusters that showed high similarity in ArcSinh5-transformed median expression of all markers were merged. To visualize phenotypic differences among the blood samples, we additionally performed t-SNE analyses on each major immune lineage where we downsampled to a maximum number of 0.5×10^6^ cells. For all analyses, frequencies of the clusters were calculated and hierarchical clustering thereof was performed using Spearman’s rank correlation in Matlab V.R2016a 9.0.

A dual t-SNE analysis was performed to quantify the similarity between individual samples based on the cluster composition.^27^ The samples t-SNE map is constructed using a data matrix (N_samples_ × K_clusters_) containing the cluster frequencies of the individual samples, hence samples with similar cluster composition profiles end up close together in the map. The data matrix was normalized by centering to zero mean and scaling to unit variance. The normalized data matrix was transposed and used to generate the clusters t-SNE map, hence clusters with similar profiles across individual samples end up close together in the map.

### Flow cytometry

Single-cell suspensions of PDAC tissues (N=5) were analyzed by flow cytometry for the expression of cytotoxic molecules and pro-inflammatory cytokines by ILC1-like cells and CD8^+^ and CD8^–^ T cells at baseline versus on stimulation. Briefly, cells were thawed and rest at 37°C in IMDM/L-glutamine medium (Lonza) complemented with 10% human serum for 1 hour. Thereafter, cells were stimulated with 20 ng/mL PMA (Sigma-Aldrich) and 1 µg/mL ionomycin (Sigma-Aldrich) for 6 hours at 37°C and then 10 μg/mL brefeldin A (Sigma-Aldrich) was added for the last 4 hours. Next, cells were washed in FACS buffer (PBS (Fresenius Kabi)/1% FCS) and incubated with human Fc receptor block (BioLegend) for 10 min at 4°C. Thereafter, cells were stained with cell surface antibodies (1:20 anti-CD7-V450 (clone M-T701, BD Biosciences), 1:20 anti-CD3-Am Cyan (clone SK7, BD Biosciences), 1:100 anti-CD8α-BV605 (clone SK1, BD Biosciences), 1:25 anti-CD127-BV711 (clone A019D5, BioLegend), 1:10 anti-CD103-FITC (clone Ber-ACT8), 1:20 anti-CD45RO-PerCP-Cy5.5 (clone UCHL1, Sony), 1:20 anti-CD45RA-PE/Dazzle (clone HI100, Sony), 1:150 anti-CD56-APC-R700 (clone NCAM16.2, BD Biosciences), and a 1:1000 nIR viability stain (Life Technologies)) for 45 min at 4°C. After two washing steps in FACS buffer (PBS/1% FCS), intracellular staining was performed using Fixation Buffer and Intracellular Staining Permeabilization Wash Buffer (BioLegend) with 1:20 anti-Perforin-PE (clone δG9, BD Biosciences), 1:50 anti-Granzyme B-PE (clone GB11, eBioscience), 1:300 anti-TNFα-PE-Cy7 (clone Mab11, eBioscience), and 1:50 anti-IFNγ-APC (clone 25723.11, BD Biosciences) for 20 min at rT, followed by three washing steps in permwash buffer. In addition, single-cell suspensions of PDAC tissues (N=7) were analyzed by flow cytometry for the expression of FOXP3 by CD25^+^CD127^low^ and CD25^+^CD127^low^ICOS^+^ CD4^+^ T cells. After incubation with human Fc receptor block, cells were stained with the following cell surface antibodies for 45 min at 4°C: 1:100 anti-CD4-BV421 (clone RPA-T4, Sony), 1:20 anti-CD45RO-PerCP-Cy5.5 (UCHL1, Sony), 1:300 anti-ICOS-PE (clone C398.4A, BioLegend), 1:150 anti-CD127-PE-Cy7 (clone AO1905, BioLegend), 1:20 anti-CD25-APC (clone 2A3, BD Biosciences), and a 1:1000 nIR viability stain (Life Technologies). After washing, cells were stained using the FOXP3 Transcription Factor Staining Buffer Set (eBioscience) with the following antibodies: 1:20 anti-FOXP3-FITC (clone PCH101, Thermo Fisher Scientific) and 1:20 anti-T-bet-BV605 (clone 4B10, BioLegend). Compensation in flow cytometry experiments was carried out with CompBeads (BD Biosciences) and ArC reactive beads (Life Technologies). Cells were acquired on a FACS LSR Fortessa 4L (BD Biosciences) running FACSDiva software V.8.0 (BD Biosciences). Data were analyzed with FlowJo software V.10.6.1 (Tree Star).

### Multispectral immunofluorescence

Multispectral immunofluorescence (IF) analysis was performed on 4 µm formalin-fixed paraffin-embedded (FFPE) tissue sections. The FFPE tissue sections were handled and stained as described previously.^28^ For the ILC panel FFPE tissue sections were deparaffinized with xylene and washed in ethanol. Heat-induced antigen retrieval in citrate buffer (10 mM, pH 6) was performed and the slides were allowed to cool down to rT. Subsequently, the tissues were blocked with Superblock buffer (Thermo Fisher Scientific) and incubated overnight at 4 degrees with the following primary antibodies: anti-CD103 (1:100 dilution, EPR4166(2)), Abcam, Cambridge, UK), anti-CD45RO (1:50 dilution, UCHL1, Cell Signalling Technology, Danvers, Massachusetts, USA). After incubation, slides were washed in PBS and detection with the following fluorescent secondary antibodies was performed: Alexa Fluor 680-labeled anti-rabbit antibody (1:400 dilution, Thermo Fisher Scientific) and CF633-labeled goat-anti-mouse IgG2a antibody (1:400 dilution, Biotium, Fremont, California, USA). After washing, tissues were incubated with directly conjugated primary antibodies: anti-CD7 (EPR4242, Abcam) labeled with Alexa Fluor 647 (1:100 dilution, Thermo Fisher Scientific) and anti-CD3 (D7A6E, Cell Signalling Technology) labeled with Alexa Fluor 594 (1:50 dilution, Thermo Fisher Scientific). Alexa Fluor labeling kits were employed to label anti-CD7 and anti-CD3 antibodies (Thermo Fisher Scientific, catalog numbers A20186 and A10239, respectively).

### Image acquisition and cell counting

For each tumor, three different regions of interest (selected by an experienced pathologist) within the tumor microenvironment were imaged at 20× magnification with the Vectra 3.0 Automated Quantitative Pathology Imaging System (Perkin Elmer). An analysis algorithm was trained manually for cell segmentation as well as immunophenotyping of cells. Cellular segmentation was performed using a counterstain-based approach with DAPI to segment nuclei and membrane markers (CD3, CD7) to detect cell contours. All images were then visually inspected for the number of CD7^+^CD3^–^CD45RO^+^CD103^+^ ILC1-like cells. For each case, cell counts were normalized by tissue area (number of cells/mm^2^).

### Whole-exome sequencing analysis

Exomes of PDAC and respective normal samples were captured using the Twist Human Core Exome kit (Twist Bioscience) and paired-end reads were sequenced on the NovaSeq 6000 system. Raw reads were then mapped with bwa-mem to the hg38 reference genome. Variant calling was done by muTect.^29^ Finally, coding variants were functionally annotated using the ensembl Variant Effect Predictor.^30^

### Statistical analysis

Cell frequencies were presented either as mean with lines indicating matched samples, as mean with SEM, or as median with IQR, as specified in the legend. Comparisons of cell frequencies between PDAC and non-malignant pancreatic tissue were performed by Mann-Whitney tests, as matched non-malignant samples were only available from 6 out of the total of 11 patients. Comparisons of cell frequencies between the different blood samples were performed by Wilcoxon matched-pairs signed-rank tests. Statistical tests were conducted in GraphPad Prism (V.9.0.1) and p values<0.05 were considered statistically significant.

## Results

### The PDAC immune microenvironment is enriched for B cells and regulatory T cells

**Figure 1:**
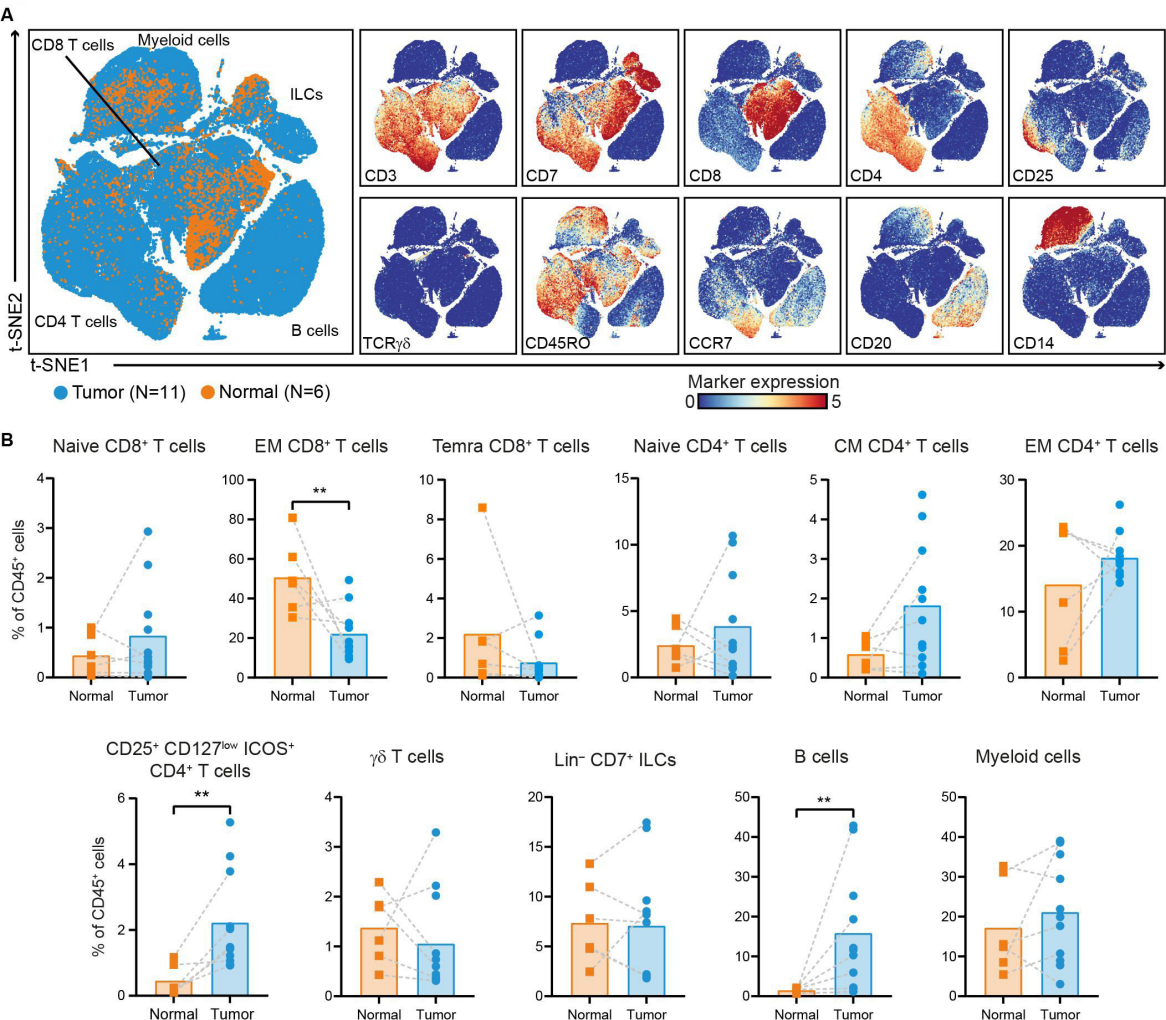
The PDAC immune microenvironment is enriched for B cells and regulatory T cells. (A) t-SNE embedding showing 155 433 immune cells isolated from PDAC (N=11) and non-malignant pancreatic tissues (N=6), clustered based on the expression of 41 immune cell markers by single-cell mass cytometry. In the right panels, the relative expression of indicated immune lineage markers is shown. (B) Frequencies of the major immune lineages in PDAC tissues (N=11) as compared with non-malignant pancreatic tissues (N=6) shown as percentage of total CD45^+^ cells. Each dot represents an individual sample. Bars indicate the mean and lines indicate matched samples. Data from 11 independent experiments with mass cytometry. **p<0.01 by Mann-Whitney test. ILCs, innate lymphoid cells; PDAC, pancreatic ductal adenocarcinoma; t-SNE, t-distributed stochastic neighbor embedding.

The immune composition of PDAC and non-malignant pancreatic tissues, matched regional lymph nodes, spleen, portal vein blood, and peripheral blood samples (collected before and after surgery) from 11 patients with PDAC was deciphered by measuring 41 immune cell markers by single-cell mass cytometry (online supplemental figure 1). HSNE analysis was performed on all acquired CD45^+^ cells (18.2×10^6^ cells in total), and showed the presence of eight major immune cell clusters (online supplemental figure 2). First, we specifically explored the immune landscape in PDAC and non-malignant pancreatic tissues by single-cell mass cytometric analysis of 11 PDAC tissues with 6 matched non-malignant pancreatic tissues. This analysis showed the presence of different clusters of CD4^+^ T cells, CD8^+^ T cells/γδ T cells, ILCs, B cells, and myeloid cells (figure 1A, online supplemental figure 3). In non-malignant pancreatic tissue, memory CD8^+^ T cells were the most prevalent subset, followed by myeloid cells, and memory CD4^+^ T cells, although the absolute number of immune cells was generally low (figure 1B, online supplemental figure 3). Compared with non-malignant tissues, PDAC tissues showed a higher relative frequency of B cells and CD4^+^CD25^+^CD127^low^ICOS^+^ cells (p<0.01 by Mann-Whitney tests; figure 1B). By flow cytometry, we demonstrated that this population was mainly comprised of FOXP3^+^, regulatory T cells (online supplemental figure 4), in line with our previous observations in colorectal cancer.^31^ In contrast, PDAC tissues showed a remarkable decreased frequency of CD8^+^ T cells with an effector memory phenotype as compared with non-malignant tissue (p<0.01 by Mann-Whitney test; figure 1B).

### PDAC tissues are largely deprived of infiltration by CD8^+^ T cells with cytotoxic potential

**Figure 2:**
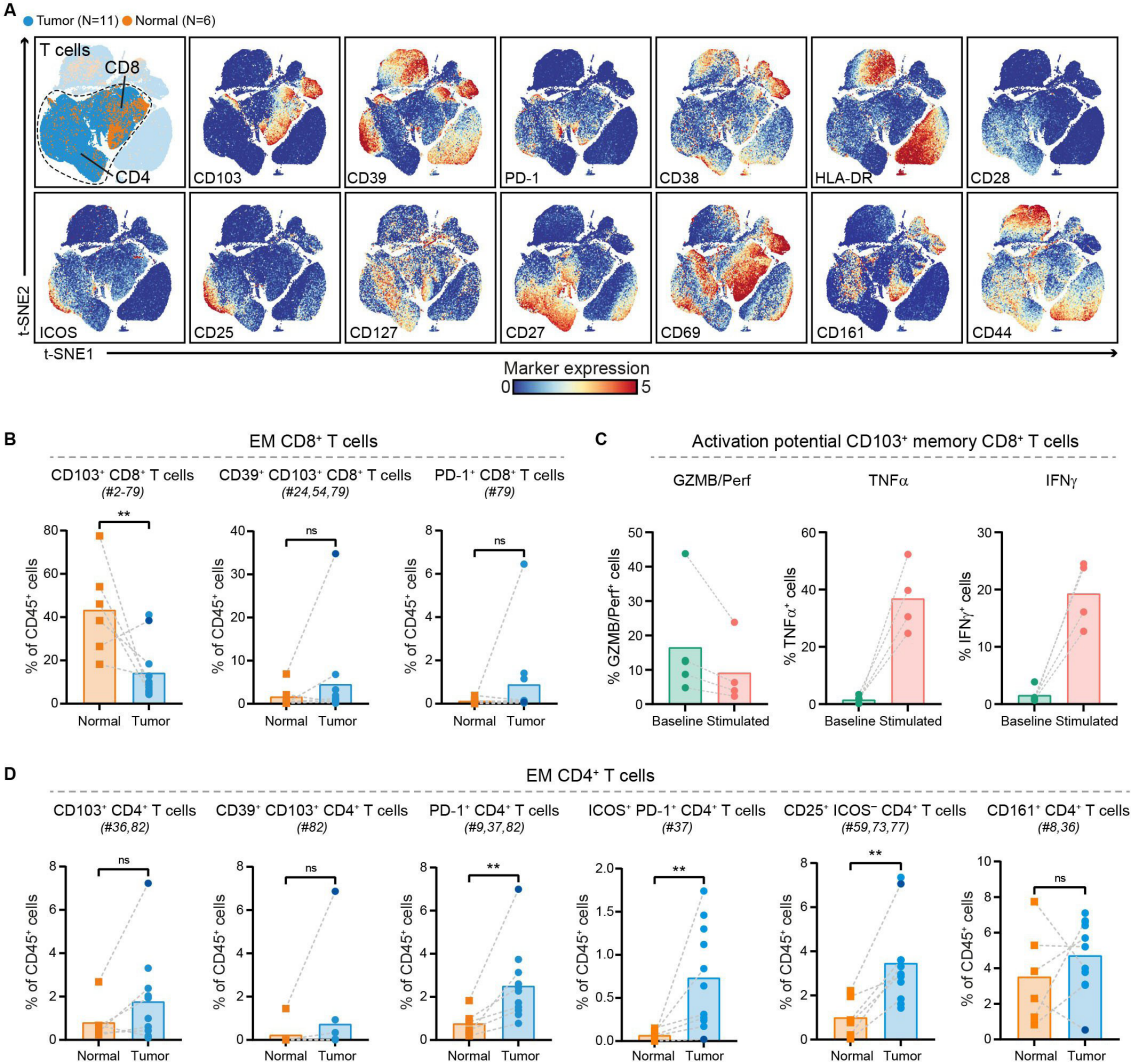
PDAC tissues are largely deprived of infiltration by CD8^+^ T cells with cytotoxic potential. (A) t-SNE embedding from figure 1A highlighting the CD8^+^ and CD4^+^ T cell populations, colored by tissue type (first plot) and relative expression of indicated T cell markers (remainder). An overview of all T cell clusters identified is shown in online supplemental figure 5. (B) Frequencies of selected effector memory CD8^+^ T cell clusters in PDAC (N=11) and non-malignant pancreatic (N=6) tissues shown as percentage of total CD45^+^ cells. (C) Cytokine production by CD103^+^ (tissue-resident) memory CD8^+^ T cells from PDAC tissues ex vivo (N=5) and on stimulation with PMA/ionomycin (n=4) measured by flow cytometry. Bars indicate the mean and lines indicate matched samples. (D) Frequencies of selected effector memory CD4^+^ T cell clusters in PDAC (N=11) and non-malignant pancreatic (N=6) tissues shown as percentage of total CD45^+^ cells. (B, D) Cluster IDs correspond to the ones in online supplemental figure 5. Each dot represents an individual sample (dark blue color represents patient ISPIC20). Bars indicate the mean and lines indicate matched samples. Data from 11 independent experiments with mass cytometry. **p<0.01 by Mann-Whitney test. GZMB/Perf, granzyme B/perforin; IFN, interferon; PDAC, pancreatic ductal adenocarcinoma; TNF, tumor necrosis factor; t-SNE, t-distributed stochastic neighbor embedding.

We further investigated the phenotype of CD8^+^ and CD4^+^ T cells in PDAC and non-malignant pancreatic tissues (figure 2A, online supplemental figure 5). Within the effector memory CD8^+^ T cells, a reduced relative frequency in CD103^+^ (tissue-residency marker) CD8^+^ T cells was observed in PDAC tissues as compared with their matched non-malignant tissues (p<0.01 by Mann-Whitney test; figure 2B), with the exception of one patient (ISPIC20; indicated by the dark blue dot). The majority of CD8^+^ T cells contained in PDAC tissues did not show expression of activation markers such as CD38, HLA-DR, and CD39 (figure 2A). The CD103^+^ memory CD8^+^ T cells contained in PDAC tissues also carried low amounts of cytolytic enzymes granzyme B/perforin and of pro-inflammatory cytokines tumor necrosis factor (TNF)α and interferon (IFN)γ (figure 2C). However, the CD103^+^ memory CD8^+^ T cells still had the potential to become activated as determined by their ability to produce TNFα and IFNγ on stimulation (figure 2C). Co-expression of CD39 and CD103 has been proposed to identify tumor-reactive CD8^+^ T cells in different solid malignancies.^32 33^ Of the 11 patients studied here, we only found CD39^+^CD103^+^CD8^+^ T cells in one PDAC tissue (ISPIC20), constituting 35% of all CD45^+^ cells in this patient (figure 2B). Interestingly, the pancreatic tumor of patient ISPIC20 showed a relatively higher number of coding, non-synonymous, somatic mutations (N=70) as compared with the rest of the cohort (average N=29, SD ±16). Whole-exome sequencing of the tumor tissue of ISPIC20 revealed the presence of a homozygous, somatic mutation in *EXO1*, involved in DNA MMR and homologous recombination, which potentially underlies the higher mutational load observed in this sample. In addition to investigating the presence of T cells with tumor-reactive phenotypes, we determined whether T cells in PDAC tissues expressed the immune checkpoint blockade molecule PD-1. We found that PD-1^+^CD8^+^ T cells were almost completely absent in the tumor tissues, with the exception of one patient (ISPIC22).

In contrast to the absence of cytotoxic T cells in the pancreatic tumors, CD4^+^ T cells were relatively abundant and showed expression of activation markers (figure 2A). CD4^+^ T cell phenotypes contained in PDAC tissues included PD-1^+^CD4^+^ T cells, of which the relative frequency was significantly increased in pancreatic tumors as compared with the non-malignant tissues (p<0.01 by Mann-Whitney test; figure 2D). A subset of these PD-1^+^CD4^+^ T cells co-expressed ICOS and this population was almost exclusively found in PDAC tissues (p<0.01 by Mann-Whitney test; figure 2D). In line with the CD8^+^ compartment, memory CD4^+^ T cells in PDAC tissues showed low levels of granzyme B/perforin, TNFα, and IFNγ ex vivo (online supplemental figure 6). Interestingly, the PDAC tissue of patient ISPIC20 showed the most CD103^+^CD4^+^ T cells as well as CD39^+^CD103^+^CD4^+^ T cells, reflecting the characteristics of the CD8^+^ compartment (figure 2D). Last, a population of CD25^+^ activated CD4^+^ T cells showed an increased relative frequency in PDAC as compared with non-malignant tissues (p<0.01 by Mann-Whitney test; figure 2D). Altogether, these data demonstrate a lack of activated, CD103^+^ cytotoxic T cells in the immune microenvironment of PDAC, while an adequate profile for the generation of helper T cell responses may be present.

### Activated, CD103^+^ ILC1-like cells are prevalent in pancreatic tumors

**Figure 3:**
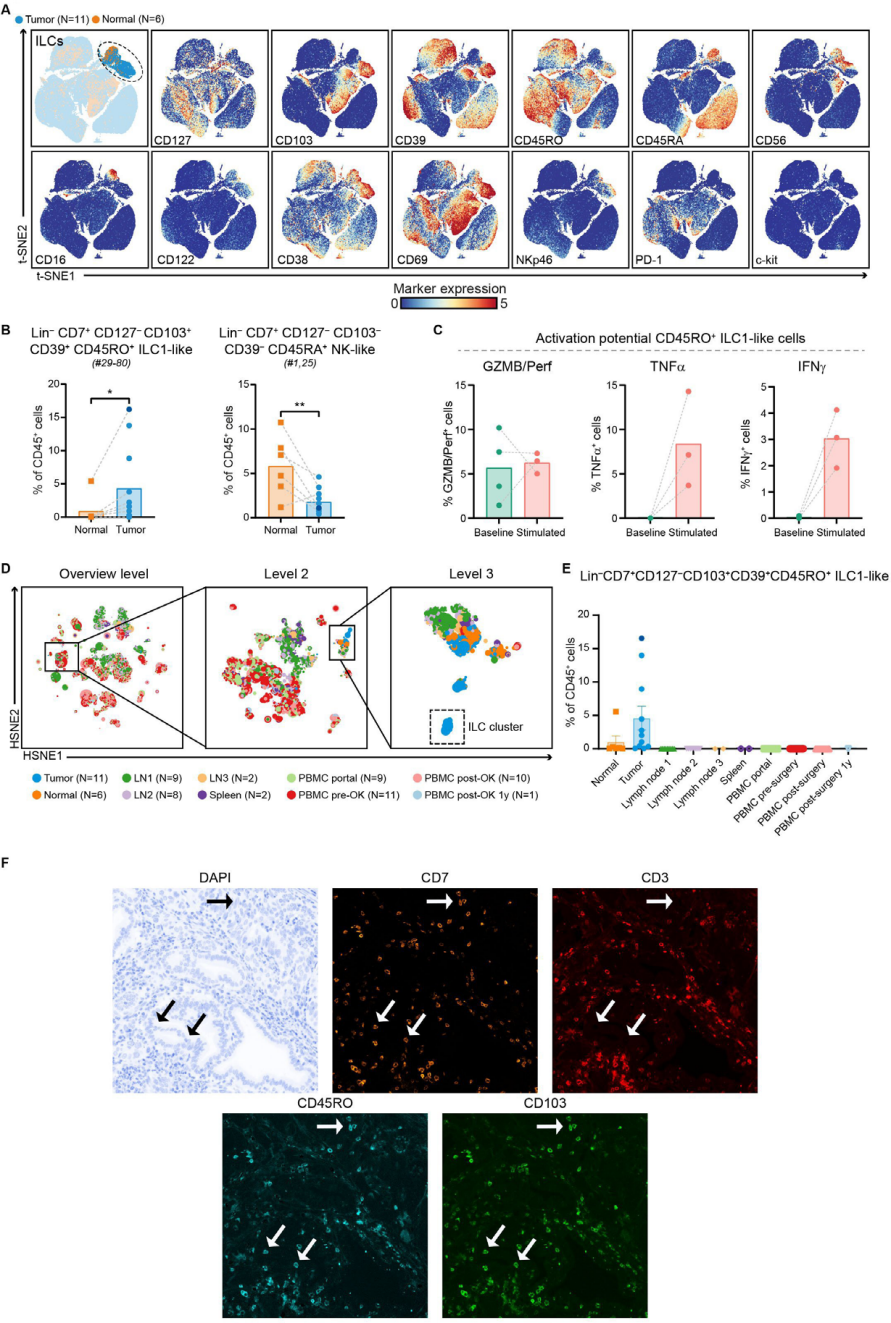
Activated, CD103^+^ ILC1-like cells are prevalent in pancreatic tumors. (A) t-SNE embedding from figure 1A highlighting the Lin^–^CD7^+^ ILC population, colored by tissue type (first plot) and relative expression of indicated ILC markers (remainder). An overview of all ILC clusters identified is shown in online supplemental figure 5. (B) Frequencies of selected ILC clusters in PDAC tissues (N=11) as compared with non-malignant pancreatic tissues (N=6) shown as percentage of total CD45^+^ cells. Cluster IDs correspond to the ones in online supplemental figure 5. Each dot represents an individual sample (dark blue color represents patient ISPIC20). Bars indicate the mean and lines indicate matched samples. Data from 11 independent experiments with mass cytometry. *P<0.05, **p<0.01 by Mann-Whitney test. (C) Cytokine production by CD45RO^+^ ILC1-like cells from PDAC tissues ex vivo (N=4) and on stimulation with PMA/ionomycin (N=3) measured by flow cytometry. Bars indicate the mean and lines indicate matched samples. (D) HSNE analysis showing 17 592 landmarks representing 18.2×10^6^ immune cells isolated from PDAC and non-malignant pancreatic tissues, regional lymph nodes, spleen, portal vein blood, and peripheral blood obtained before and after surgery from 11 patients with PDAC clustered based on the expression of 41 immune cell markers by CyTOF. Specific landmarks were selected and embedded at the next, more detailed levels to identify the CD127^–^CD103^+^CD39^+^CD45RO^+^ ILC1-like cells and their distribution across the different tissue types. Colors represent the different tissue types. (E) Frequencies of CD127^–^CD103^+^CD39^+^CD45RO^+^ ILC1-like cells among non-malignant pancreatic (N=6) and PDAC (N=11) tissues, regional lymph nodes (details below), spleen (N=2), portal vein blood (N=8), peripheral blood before surgery (N=11), directly after surgery (N=10), and 1 year after surgery (N=1) from 11 patients with PDAC shown as percentage of total CD45^+^ cells. Each dot represents an individual sample. Bars indicate the mean with SEM. Data from 11 independent experiments with mass cytometry. Lymph node 1 derived from the common hepatic artery (N=9), lymph node 2 from the hepatoduodenal ligament (N=8), and lymph node 3 from the abdominal aorta (N=2). (F) Representative images of the immunofluorescence microscopic detection of CD7^+^CD3^–^CD103^+^CD45RO^+^ ILC1-like cells in a pancreatic tumor. GZMB/Perf, granzyme B/perforin; HSNE, hierarchical stochastic neighbor embedding; IFN, interferon; ILCs, innate lymphoid cells; PBMC, peripheral blood mononuclear cells; PDAC, pancreatic ductal adenocarcinoma; TNF, tumor necrosis factor; t-SNE, t-distributed stochastic neighbor embedding.

Detailed analysis of Lin^–^CD7^+^ ILCs in PDAC and non-malignant pancreatic tissue revealed the presence of a largely tumor tissue-specific ILC population, characterized by the lack of conventional ILC marker CD127 and the expression of CD103, CD39 and CD45RO (figure 3A, online supplemental figure 5). These CD127^–^CD103^+^CD39^+^CD45RO^+^ ILCs constituted up to 16% of the immune cell infiltrate in pancreatic tumors (figure 3B), and were present at a higher relative frequency than in non-malignant tissue (p<0.05 by Mann-Whitney test; figure 3B). Interestingly, we previously found that such CD127^–^CD103^+^CD45RO^+^ ILCs are particularly enriched in MMR-deficient colorectal cancers, and have characteristics of intraepithelial ILC1-like cells.^31^ In contrast to the exceptional high levels of cytotoxic enzymes present in this population in colorectal cancers, we found that only up to 10% of the CD45RO^+^ ILC1-like cells expressed granzyme B/perforin in PDAC tissues. When stimulated, low levels of TNFα and IFNγ were also produced by the CD45RO^+^ ILC1-like cells, up to 14% and 4%, respectively (figure 3C, online supplemental figure 7). In non-malignant tissues, CD127^–^CD103^–^CD39^–^CD45RA^+^ NK-like cells were the most frequent ILC population (figure 3B, online supplemental figure 5).

We subsequently examined whether the ILC1-like population was also present in regional lymph nodes, spleen, portal vein blood, or peripheral blood by running an HSNE analysis on all samples from the patients with PDAC (figure 3D). This analysis confirmed the specific localization within the tumor tissue of the CD127^–^CD103^+^CD39^+^CD45RO^+^ ILC1-like population, as the cells were not found in regional lymph nodes, spleen, portal vein blood, or peripheral blood (figure 3E). To determine the spatial localization of the ILC1-like cells in PDAC tissues, a 5-color multispectral IF panel was used on FFPEs tissue sections of the pancreatic tumors. We identified a high proportion of CD7^+^CD3^–^CD45RO^+^CD103^+^ ILC1-like cells in the immune microenvironment of the tumors, in line with the CyTOF data (figure 3F). The ILC1-like cells frequently localized adjacent to the ductal cells, which is in agreement with their CD103^+^ tissue-resident phenotype. Thus, we discovered an activated, CD103^+^ ILC1-like population specifically enriched in pancreatic cancers.

### Increased frequencies of CD27^+^ memory B cells in PDAC

**Figure 4:**
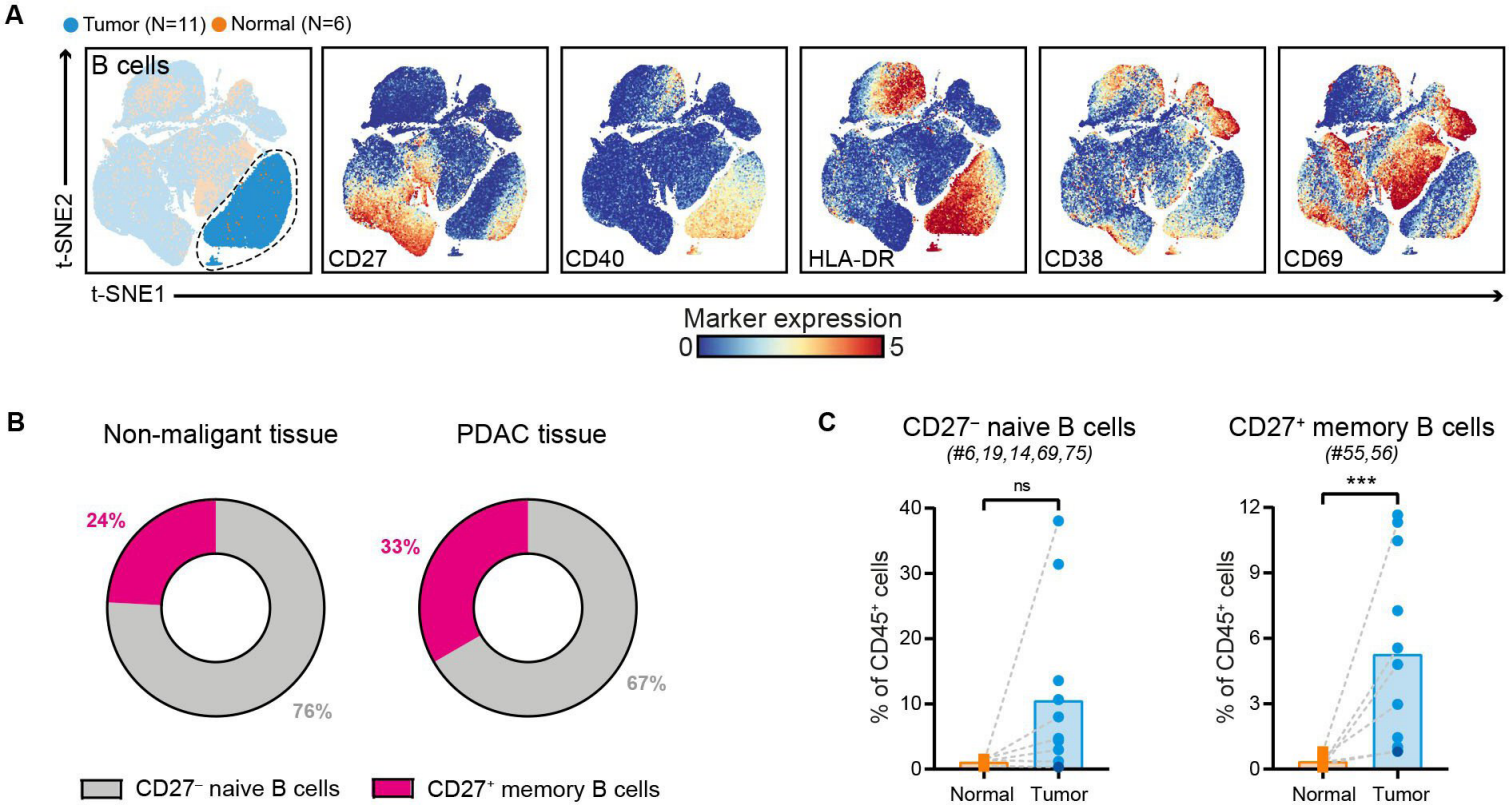
Increased frequencies of CD27^+^ memory B cells in PDAC. (A) t-SNE embedding from figure 1A highlighting the CD20^+^ B cells, colored by tissue type (first plot) and relative expression of indicated B cell markers (remainder). An overview of all B cell clusters identified is shown in online supplemental figure 5. (B) Circular plots showing the relative frequency of CD27^–^ naïve B cells and CD27^+^ memory B cells as average percentage of total B cells in non-malignant (N=6) and PDAC tissues (N=11). (C.) Frequencies of CD27^–^ naive B cells and CD27^+^ memory B cells in PDAC tissues as compared with non-malignant pancreatic tissues shown as percentage of total CD45^+^ cells. Cluster IDs correspond to the ones in online supplemental figure 5. Each dot represents an individual sample (dark blue color represents patient ISPIC20). Bars indicate the mean and lines indicate matched samples. Data from 11 independent experiments with mass cytometry. ***p<0.001 by Mann-Whitney test. PDAC, pancreatic ductal adenocarcinoma; t-SNE, t-distributed stochastic neighbor embedding.

Immune marker profiles of the B cell compartment showed that CD27^–^ naïve B cells were predominant in PDAC tissues (accounting for 67% of the B cell population), followed by CD27^+^ memory B cells (accounting for 33% of the B cell population) (figure 4A, B, online supplemental figure 5). Compared with non-malignant tissues, the relative frequencies of both B cell subsets were increased in PDAC tissues (CD27^+^ memory B cells: p<0.001 by Mann-Whitney test; figure 4C). Importantly, none of the tumor samples with higher B cell frequency showed signs of pancreatitis or contained tertiary lymphoid structures (TLS) as confirmed by histological examination of the PDAC tissues. Both CD27^–^ naïve B cells and CD27^+^ memory B cells in PDAC tissues expressed HLA-DR and CD40 (figure 4A), the latter receptor involved in mediating T cell help. In contrast, expression of activation marker CD69 was found only on the CD27^+^ memory B cell population (figure 4A). Last, examination of the composition of the myeloid compartment in PDAC and non-malignant pancreatic tissues showed no significant differences in the frequency of CD33^+^HLA-DR^low^ myeloid cells, myeloid dendritic cells (DCs), and plasmacytoid DCs (online supplemental figure 8). Intriguingly, expression of ICAM-1 and CD16 on myeloid cells was mainly observed in PDAC tissues and not in non-malignant pancreatic tissue (online supplemental figure 8).

### Portal vein blood can reflect the immune cell populations residing in pancreatic cancers

**Figure 5:**
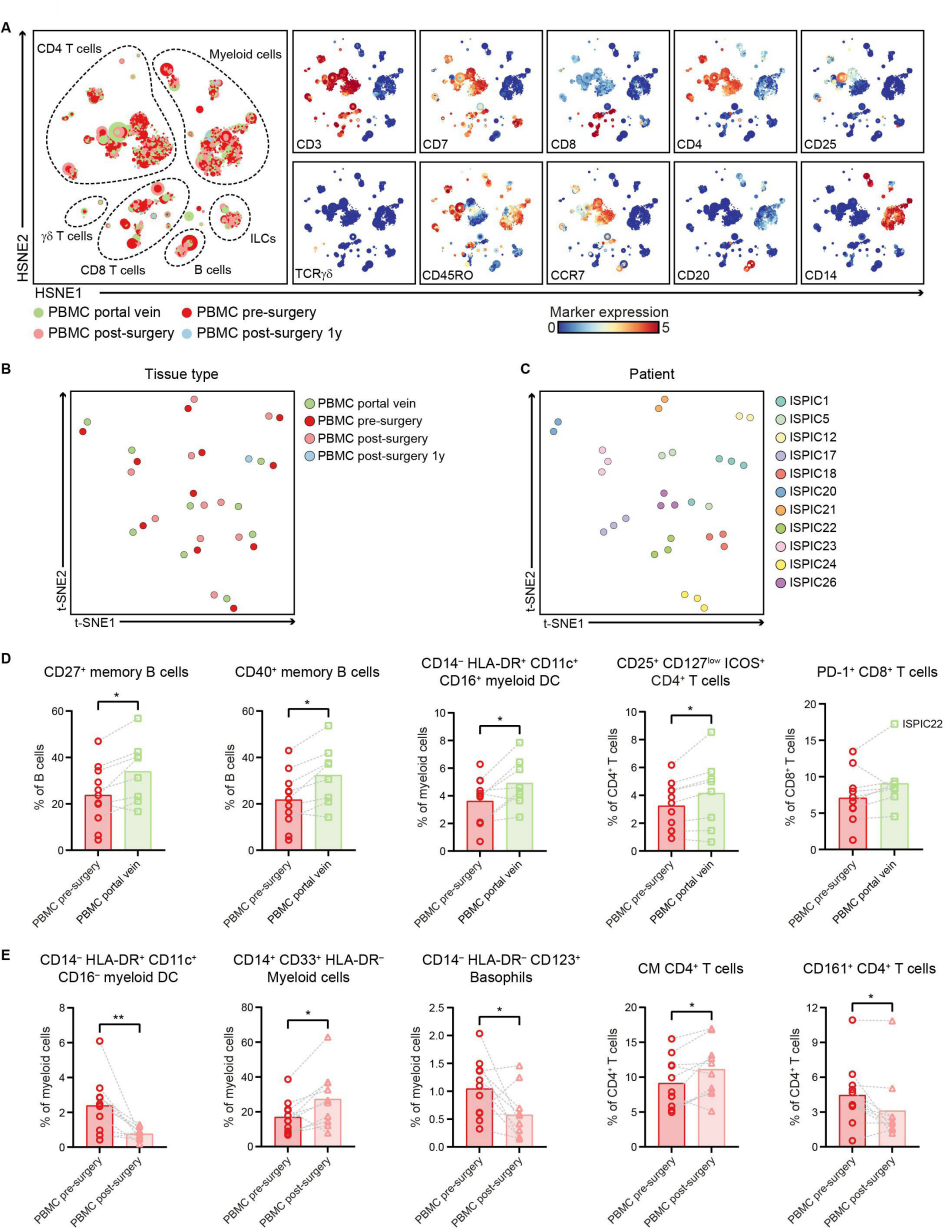
Portal vein blood can reflect the immune cell populations residing in pancreatic cancers. (A) HSNE analysis showing 9921 landmarks representing 10.9×10^6^ immune cells isolated from portal vein blood (N=8), peripheral blood before surgery (N=11), peripheral blood directly after surgery (N=10) as well as 1 year after surgery (N=1) from 11 patients with PDAC clustered based on the expression of 41 immune cell markers by single-cell mass cytometry. In the right panel, the relative expression of indicated immune lineage markers is shown. (B) Collective t-SNE analysis showing the clustering of all blood samples from the patients with PDAC (N=31) based on cell percentage data (of CD45^+^ cells) of all immune cell clusters identified (N=417). Every dot represents a sample colored by blood type. (C) Collective t-SNE analysis of (B) colored by patient ID. (D) Frequencies of selected immune cell populations in portal vein blood (N=8) and peripheral blood pre-surgery (N=11) as percentage of the indicated major immune lineage. (E) Frequencies of selected immune cell populations in peripheral blood pre-surgery (N=11) and postsurgery (N=10) as percentage of the indicated major immune lineage. (D, E) Each dot represents an individual sample. Bars indicate the mean and lines indicate matched samples. Data from 11 independent experiments with mass cytometry. *p<0.05, **p<0.01 by Wilcoxon test. HSNE, hierarchical stochastic neighbor embedding; ILCs, innate lymphoid cells; PBMC, peripheral blood mononuclear cells; PDAC, pancreatic ductal adenocarcinoma; t-SNE, t-distributed stochastic neighbor embedding.

In addition to characterizing the immune infiltration in PDAC and non-malignant pancreatic tissues, we examined the systemic immune profiles of patients with PDAC through analysis of different blood samples collected from (1) portal vein blood, (2) peripheral blood before surgery, and (3) peripheral blood after surgery. HSNE analysis of all immune cells showed an equivalent distribution of the major immune lineages across the different blood sample types with large populations of myeloid cells and CD4^+^ T cells (figure 5A, online supplemental figure 9), except for an increase in the relative frequency of γδ T cells in portal vein blood as compared with the matched peripheral blood samples (p<0.05 by Wilcoxon test; online supplemental figure 9). The clustering of all blood samples based on their complete immune composition showed that inter-patient heterogeneity was greater than potential differences in immune cell profiles between the different compartments or time points, per patient (figure 5B,C). As such, we performed an analysis per immune lineage to investigate phenotypic differences between the different types of blood samples from patients with PDAC.

Strikingly, increased frequencies of the immune cell populations enriched in PDAC tissue were also found in portal vein blood as compared with peripheral blood. For example, in portal vein blood, an increased frequency of CD27^+^ memory B cells was found as compared with peripheral blood (p<0.05 by Wilcoxon test; figure 5D, online supplemental figure 10), similar to the increased presence of memory B cells observed in the PDAC tissues (figure 4C). The large majority of the CD27^+^ memory B cells in portal blood also expressed CD40 (figure 5D, online supplemental figure 10). Besides, portal vein blood contained higher frequencies of HLA-DR^+^CD11c^+^CD16^+^ myeloid DCs as compared with presurgery peripheral blood (p<0.05 by Wilcoxon test; figure 5D). Moreover, portal vein blood showed a higher relative frequency of CD4^+^CD25^+^CD127^low^ICOS^+^ regulatory T cells as compared with presurgery peripheral blood (p<0.05 by Wilcoxon test; figure 5D, online supplemental figure 11), in line with the increased presence of regulatory T cells found in the PDAC tissues (figure 2B). Of note, the only patient that showed infiltration of PD-1^+^CD8^+^ T cells in the tumor (ISPIC22), showed the highest levels of PD-1^+^CD8^+^ T cells in portal blood (17% of CD8 T cells; figure 5D), suggesting that local immune profiles in PDAC tissues can also be detected in the blood circulation close to the tumor.

When comparing peripheral blood collected before and after surgery, we observed a decreased frequency of CD16^–^ myeloid DCs in postsurgery versus presurgery peripheral blood (p<0.01 by Wilcoxon test; figure 5E, online supplemental figure 10). This was accompanied by an increase in CD14^+^CD33^+^HLA-DR^–^ myeloid cells in peripheral blood after surgery as compared with before (p<0.05 by Wilcoxon test; figure 5E). A higher proportion of myeloid-derived suppressor cells (MDSCs) relative to the myeloid DCs has been previously described and suggested to play a role in the clinical recovery of patients following surgical procedure.^34^ Last, a population of CD14^–^HLA-DR^–^CD123^+^ basophils was substantially lower in peripheral blood after surgery compared with before (p<0.05 by Wilcoxon test; figure 5E). Analysis of the CD4^+^ T cell compartment in blood showed an increase in CD4^+^ T cells with a central memory phenotype in peripheral blood after surgery as compared with before (p<0.05 by Wilcoxon test; figure 5E), while no differences were observed in the frequencies of naïve and effector memory CD4^+^ T cells (online supplemental figure 11). Last, peripheral blood obtained after surgery showed a reduction in the number of CD161^+^CD4^+^ T cells (p<0.05 by Wilcoxon test; figure 5E), a subset of CD4^+^ T cells described to possess enhanced innate-like cytokine production.^35^

## Discussion

By studying the innate and adaptive immune composition of PDAC and matched non-malignant pancreatic tissues, regional lymph nodes, portal vein blood, and peripheral blood before and after surgery from treatment-naïve patients with PDAC, our work provides an in-depth immune characterization of local and systemic immune profiles in patients with PDAC.

Although it has been shown that CD8^+^ T cells in proximity to PDAC cells correlate with increased survival,^36^ we found that memory CD8^+^ T cells were less frequent in pancreatic cancers in comparison to non-malignant pancreatic tissue. Moreover, the few CD8^+^ T cells contained in the tumors showed a lack of tissue-residency and activation markers, cytolytic enzymes, and immune checkpoint molecules, hallmarks associated with enhanced efficacy of immune checkpoint blockade therapy. In contrast to Steele *et al* that reported heterogenous expression of immune checkpoints in PDAC tissues,^15^ we showed that the majority of the CD8^+^ T cells lacked expression of immune checkpoint molecules ICOS, CD28, CD27, and PD-1. The low amount of infiltrating PD-1 expressing immune cells in PDAC tissues was recently confirmed by conventional immunohistochemistry.^9^ Moreover, CD8^+^ and CD4^+^ T cells co-expressing CD39 and CD103 were only observed in one patient that showed a higher mutational load than the remaining samples, suggesting an absence of tumor-reactive T cells in the majority of pancreatic tumors. Finally, and in line with other studies, we found that γδ T cells comprised a small proportion of all T cells in PDAC.^9 15^

B cells and CD4^+^CD25^+^CD127^low^ICOS^+^ cells, identified as being mainly comprised of regulatory T cells,^31^ were more frequent in PDAC tissues as compared with non-malignant pancreatic tissues. The role of B cells in PDAC remains controversial. Murine studies have reported on tumor-promoting roles for B cells,^37–39^ whereas human PDAC data indicated that tumor-infiltrating B cells are a positive prognostic factor.^40 41^ Especially the location of B cells in TLS was correlated with a longer survival.^40^ In our cohort of pancreatic tumors, the increase in B cells was not related to the presence of TLS. The high relative frequency of regulatory T cells in PDAC is consistent with previous observations in mouse models and human, and in agreement with the notion that regulatory T cells, together with myeloid cells, are key immunosuppressive components in PDAC.^42–45^ More importantly, the regulatory T cells in pancreatic tumors expressed high levels of ICOS, which highlights the potential use of ICOS antagonists to inhibit Treg interactions with ICOSL (such as NCT03829501).

Within the innate compartment, we discovered a previously unappreciated ILC1-like population, CD127^–^CD103^+^CD39^+^CD45RO^+^, that was specifically found in pancreatic cancers and not in matched regional lymph nodes, spleen, portal vein blood, or peripheral blood. Interestingly, these cells resembled ILC1-like cells found in colorectal cancer expressing high levels of granzyme B and perforin.^31^ Here, we found that the ILC1-like cells produced lower amounts of cytolytic enzymes, possibly related to the highly immunosuppressive microenvironment in PDAC. Importantly, these ILCs did not express the immune checkpoint molecule PD-1, in contrast to a recent study reporting on the existence of PD-1^+^ ILC2 in mouse models of PDAC.^46^ As opposed to the CD103^+^CD39^+^CD45RO^+^ ILC1-like cells in PDAC tissues, ILCs in the matched non-malignant pancreatic tissue demonstrated a CD103^–^CD39^–^CD45RA^+^ NK-like phenotype. Further research needs to elucidate the specific mechanism by which these cells infiltrate or differentiate in PDAC; whether the ILCs acquire the CD103^+^CD39^+^CD45RO^+^ phenotype locally in PDAC tissues differentiating from CD103^–^CD39^–^CD45RA^+^ NK cells, or whether they are already CD45RO^+^ but acquire markers of tumor-residence and activation (CD103/CD39) later.

A unique feature of this study is the sample collection of portal vein blood in addition to peripheral blood before and after surgery. Intriguingly, we found that immune profiles of portal vein blood mirrors the increased frequency of memory B cells and regulatory T cells in pancreatic cancers, suggesting a regional enrichment of immune cells involved in the antitumor immune response. Furthermore, the only patient that harbored PD-1^+^CD8^+^ T cells in the tumor, also showed the highest relative frequency of PD-1^+^CD8^+^ T cells in the matched portal blood sample. This highlights a possible novel source of T cells with tumor-reactive phenotypes. It would be of interest to study if the PD-1^+^CD8^+^ T cells found in portal vein blood include tumor-reactive T cells.

In conclusion, our results confirm the unconventional nature of the immune microenvironment in PDAC, highlighting the involvement of ILC1-like cells and B cells. Larger patient cohorts are required to determine the functional role of ILC1-like cells and B cells in pancreatic cancers and their possible contribution to immunotherapeutic response. Furthermore, the regional enrichment in portal vein blood of immune cells infiltrating the PDAC microenvironment might provide an interesting source of tumor-associated immune cells that could be exploited in the context of adoptive T cell transfer.

## Data Availability

Data are available upon reasonable request.
