# Supplementary material for: Local and systemic immune profiles of human pancreatic ductal adenocarcinoma revealed by single-cell mass cytometry
Source: J Immunother Cancer. 2022 Jul 6;10(7):e004638. doi: 10.1136/jitc-2022-004638 (PMC9260840; doi:10.1136/jitc-2022-004638)
Supplement: Supplementary data [file jitc-2022-004638supp001.pdf]

Supplemental figure 1

A

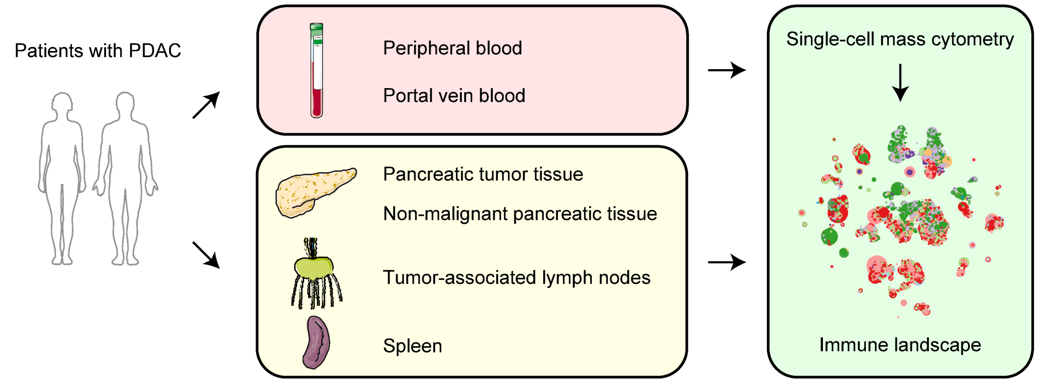

B Mass cytometry antibody panel (N = 41)

Immune lineage markers	
**CD45**	Pan immune
**CD3**	T cells
**CD4**	CD4 T cells
**CD8α**	CD8 T cells
**TCRγδ**	γδ T cells
**CD20**	B cells
**CD7**	ILCs
**CD14**	Monocytes
**CD11c**	Myeloid cells
**CD33**	MDSCs
**CD15**	Granulocytes

Fc/complement receptors	
**CD11b**	Complement R3
**CD16**	Low affinity FCγR3α

Differentiation/activation markers	
**CD45RO**	CD45 isoform
**CD45RA**	CD45 isoform
**CD38**	cADP ribose hydrolase
**CD39**	Ectonucleotidase
**CD161**	KLRB1
**HLA-DR**	Ag presentation
**CD335**	NKp46

Cytokine/chemokine receptors	
**CD25**	IL-2Rα
**CD122**	IL-2Rβ
**CD123**	IL-3Rα
**CD127**	IL-7Rα
**CCR6**	Chemokine R6
**CCR7**	Chemokine R7
**c-kit**	SCFR

Immunomodulatory molecules	
**CD27**	Co-stimulatory R
**CD28**	Co-stimulatory R
**CD40**	Co-stimulatory R
**ICOS**	Co-stimulatory R
**PD-1**	Co-inhibitory R
**PD-L1**	Co-inhibitory L

Adhesion/homing molecules	
**CD44**	Glycoprotein
**CD54**	ICAM-1
**CD56**	NCAM
**CD57**	HNK1
**CD69**	Glycoprotein
**CD103**	Glycoprotein
**CD163**	High-affinity scavenger R
**KLRG-1**	Glycoprotein

C Mass cytometry gating strategy

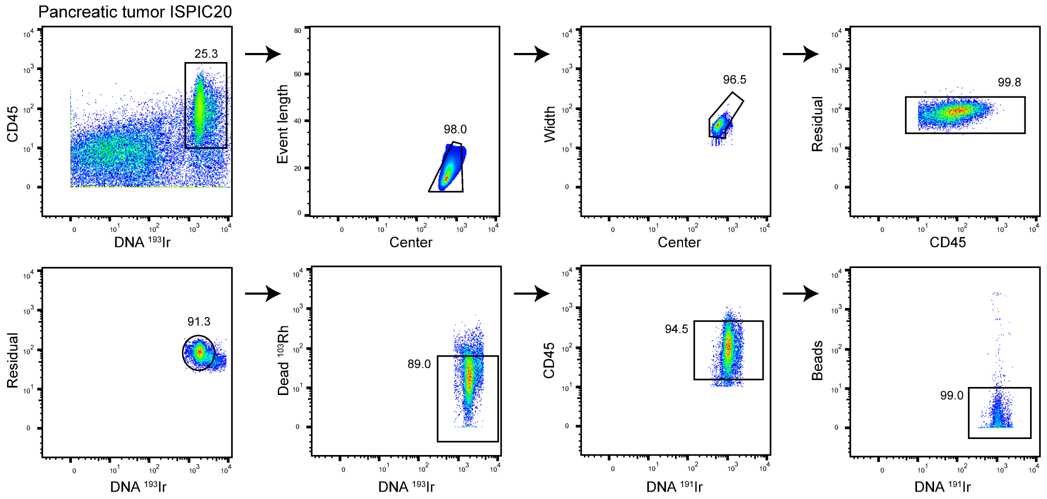

D

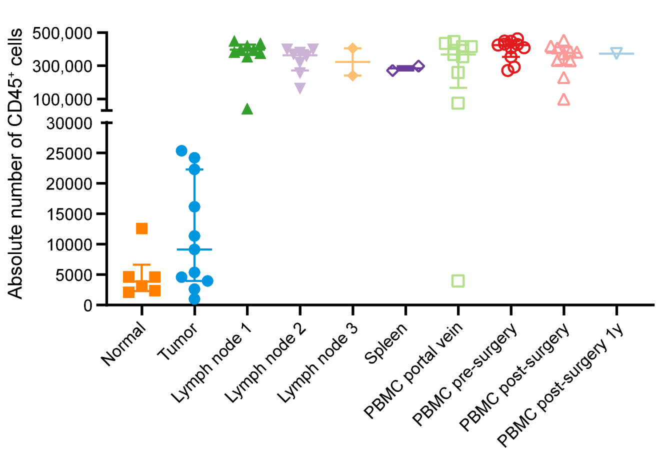

E

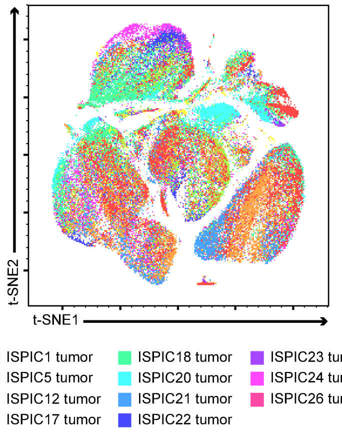

Supplemental figure 1. Overview of the study design, the mass cytometry antibody panel, and the gating strategy. A. Schematic overview of the study design. **B.** Mass cytometry antibody panel composed of 41 immune cell markers. **C.** Mass cytometry gating strategy for single, live $CD45^+$ cells of a representative pancreatic tumor sample showing sequential gates with percentages. **D.** Absolute number of $CD45^+$ cells from non-malignant pancreatic (N=6) and PDAC (N=11) tissues, regional lymph nodes (details below), spleen (N=2), portal vein blood (N=9), peripheral blood before surgery (N=11), directly after surgery (N=10), and one year after surgery (N=1) from 11 patients with PDAC. Bars indicate the median with IQR. Each dot represents an individual sample. Data from 11 independent experiments with mass cytometry. Lymph node 1 derived from the common hepatic artery (N=9), lymph node 2 from the hepatoduodenal ligament (N=8), and lymph node 3 from the abdominal aorta (N=2). **E.** t-SNE embedding of figure 1A showing the clustering of immune cells from PDAC tissues (N=11) colored by patient ID.

Supplemental figure 2

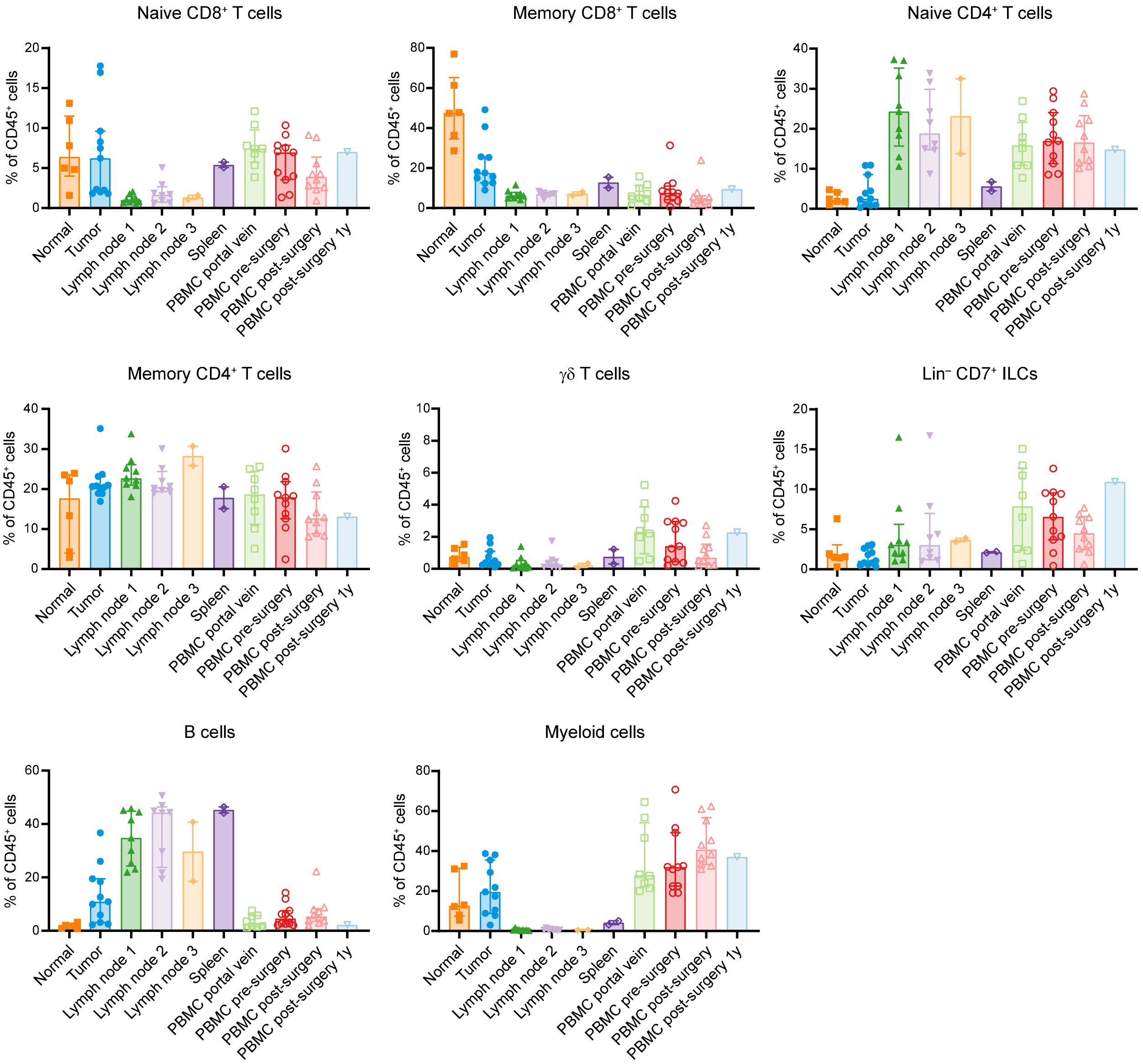

Supplemental figure 2. Frequencies of the major immune lineages in the different tissue types obtained from PDAC patients. Frequencies of the major immune lineages across non-malignant pancreatic (N=6) and PDAC (N=11) tissues, regional lymph nodes (details below), spleen (N=2), portal vein blood (N=8), peripheral blood before surgery (N=11), directly after surgery (N=10), and one year after surgery (N=1) from 11 patients with PDAC. Frequencies were determined by HSNE analysis including all samples (details in methods), and are shown as percentage of total $CD45^+$ cells. Bars indicate the median with IQR. Each dot represents an individual sample. Data from 11 independent experiments with mass cytometry. Lymph node 1 derived from the common hepatic artery (N=9), lymph node 2 from the hepatoduodenal ligament (N=8), and lymph node 3 from the abdominal aorta (N=2).

Supplemental figure 3

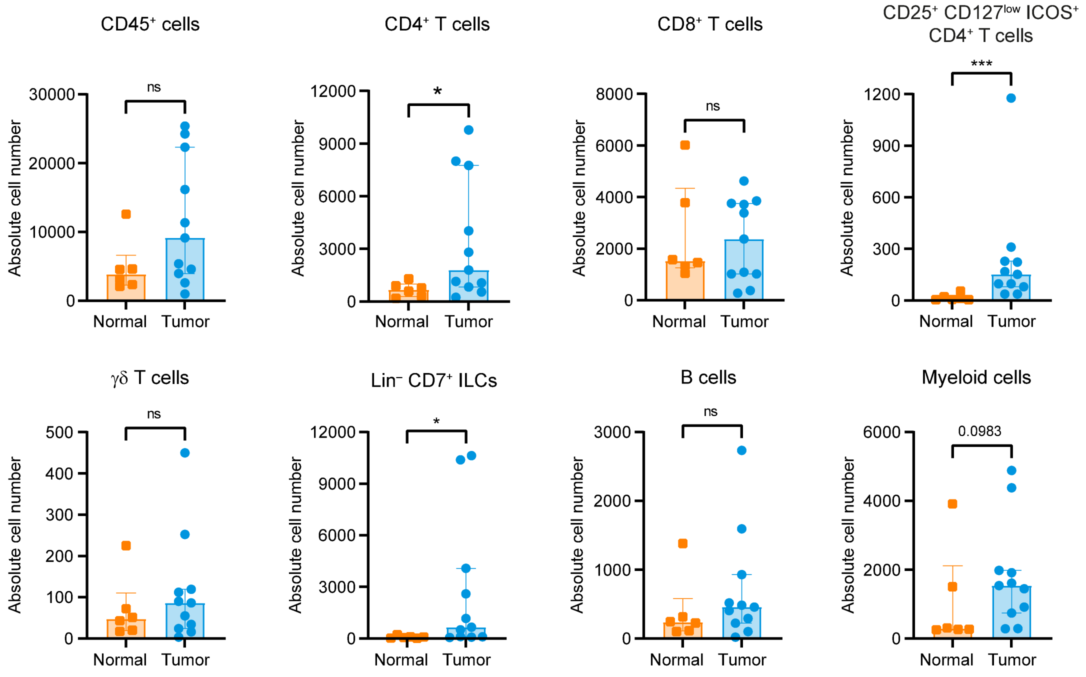

Supplemental figure 3. Absolute cell counts of the major immune lineages in PDAC and non-malignant pancreatic tissue. Absolute cell counts of the major immune lineages in non-malignant pancreatic (N=6) and PDAC (N=11) tissues as determined by mass cytometric analysis. Bars indicate the median with IQR. Data from 11 independent experiments with mass cytometry. *P<0.05, ***P<0.001 by Mann-Whitney test.

Supplemental figure 4

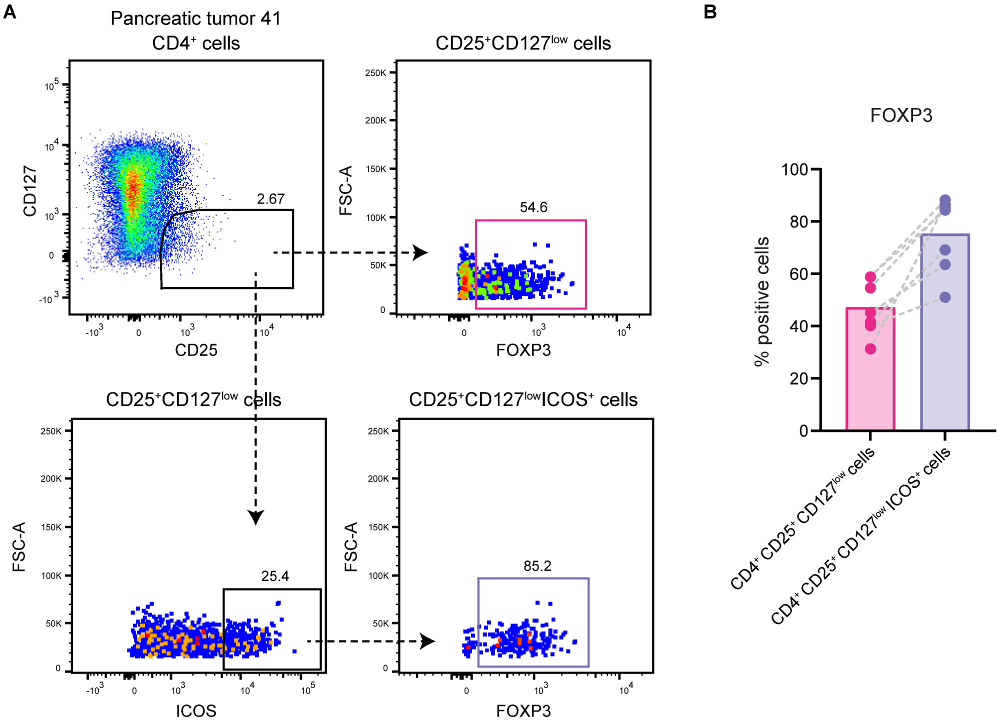

Supplemental figure 4. FOXP3 expression in CD25$^+$CD127^{low} and CD25$^+$CD127^{low}ICOS$^+$ CD4$^+$ T cells in pancreatic tumors. A. Flow cytometry gating strategy used to determine FOXP3-positive cells in a representative pancreatic tumor. **B.** Frequency of FOXP3-positive cells within CD25$^+$CD127^{low} CD4$^+$ T cells as well as within CD25$^+$CD127^{low}ICOS$^+$ CD4$^+$ T cells of pancreatic tumors (N=7).

Supplemental figure 5

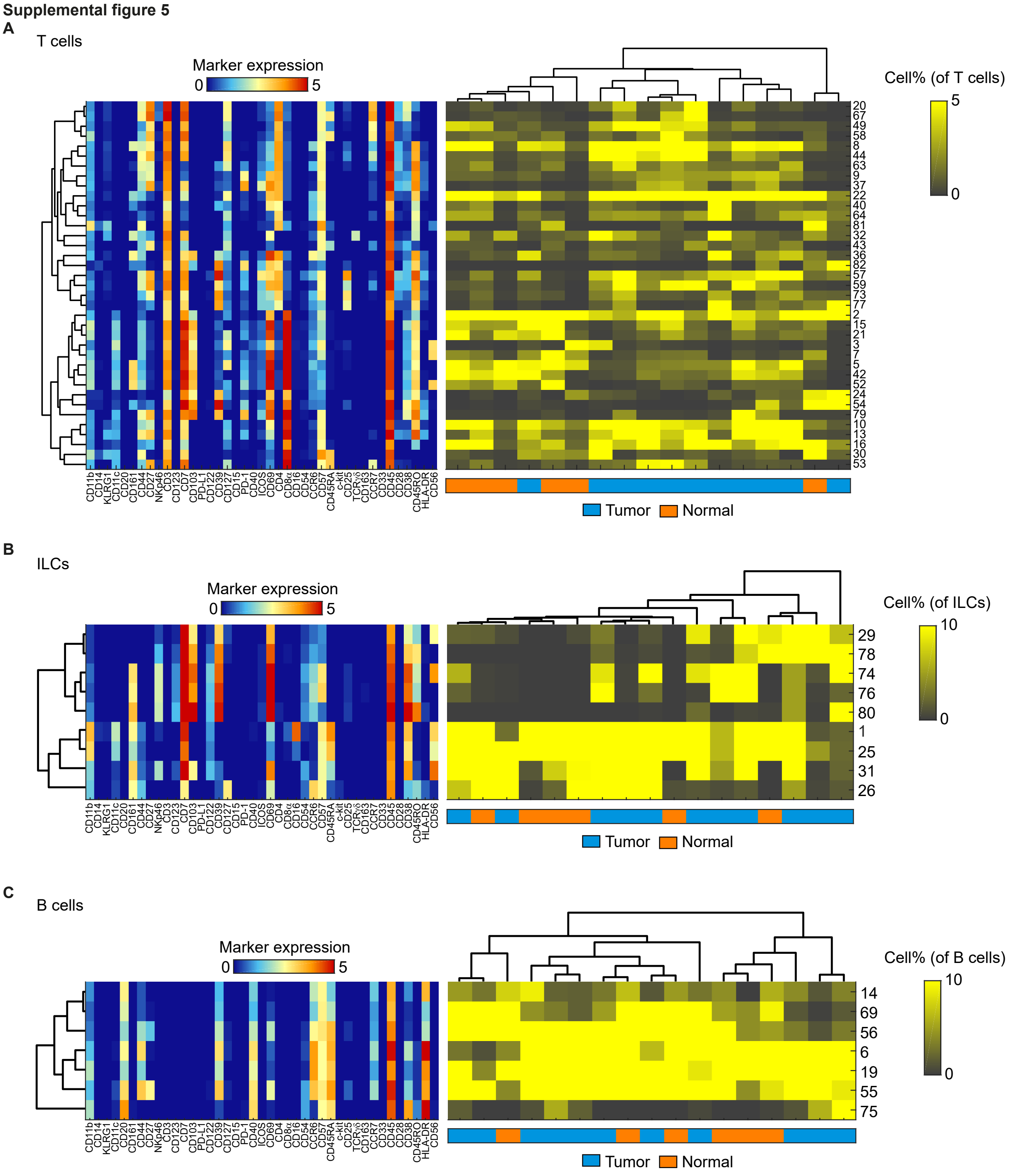

Supplemental figure 5. Marker expression and cell frequency of T cell, ILC, and B cell clusters identified in PDAC and non-malignant pancreatic tissues. A. A heatmap showing median marker expression values (left) and frequencies of all identified T cell clusters among PDAC and non-malignant pancreatic tissues (right) belonging to figure 2. **B.** A heatmap showing median marker expression values (left) and frequencies of all identified ILC clusters among PDAC and non-malignant pancreatic tissues (right) belonging to figure 3. **C.** A heatmap showing median marker expression values (left) and frequencies of all identified B cell clusters among PDAC and non-malignant pancreatic tissues (right) belonging to figure 4. Hierarchical clustering was performed on cluster frequencies using Spearman's rank correlation.

Supplemental figure 6

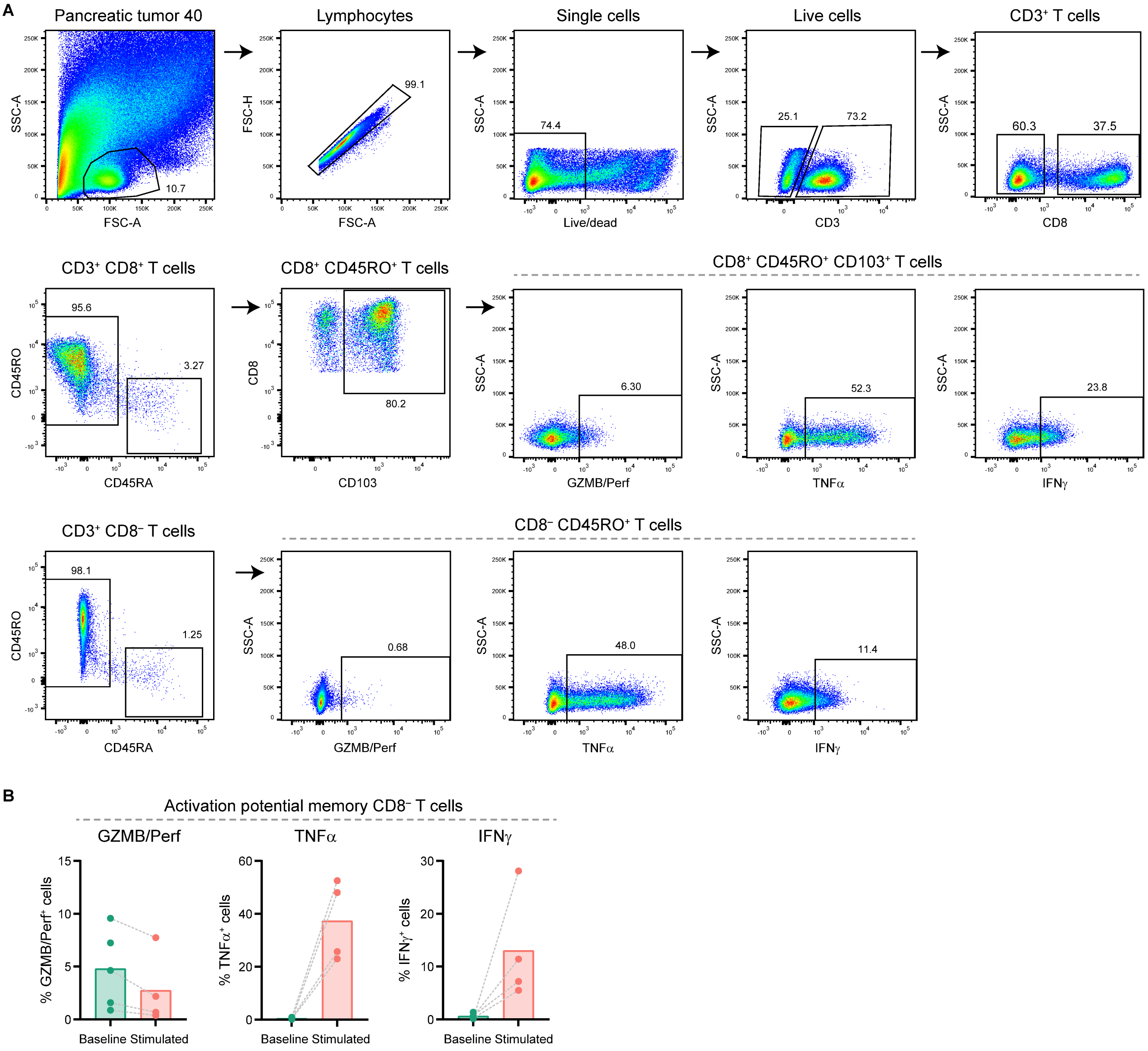

Supplemental figure 6. Gating strategy for the detection of cytotoxic molecules and cytokines by T cells. A. Flow cytometry gating strategy to detect granzyme B/perforin, TNFα, and IFNγ in CD103⁺ (tissue-resident) memory CD8⁺ T cells and in memory CD8⁻ T cells of a representative pancreatic tumor sample (upon stimulation with PMA/ionomycin) showing sequential gates with percentages. **B.** Cytokine production by memory CD8⁻ T cells from PDAC tissues *ex vivo* (N=5) and upon stimulation with PMA/ionomycin (N=4) measured by flow cytometry. Bars indicate the mean and lines indicate matched samples.

Supplemental figure 7

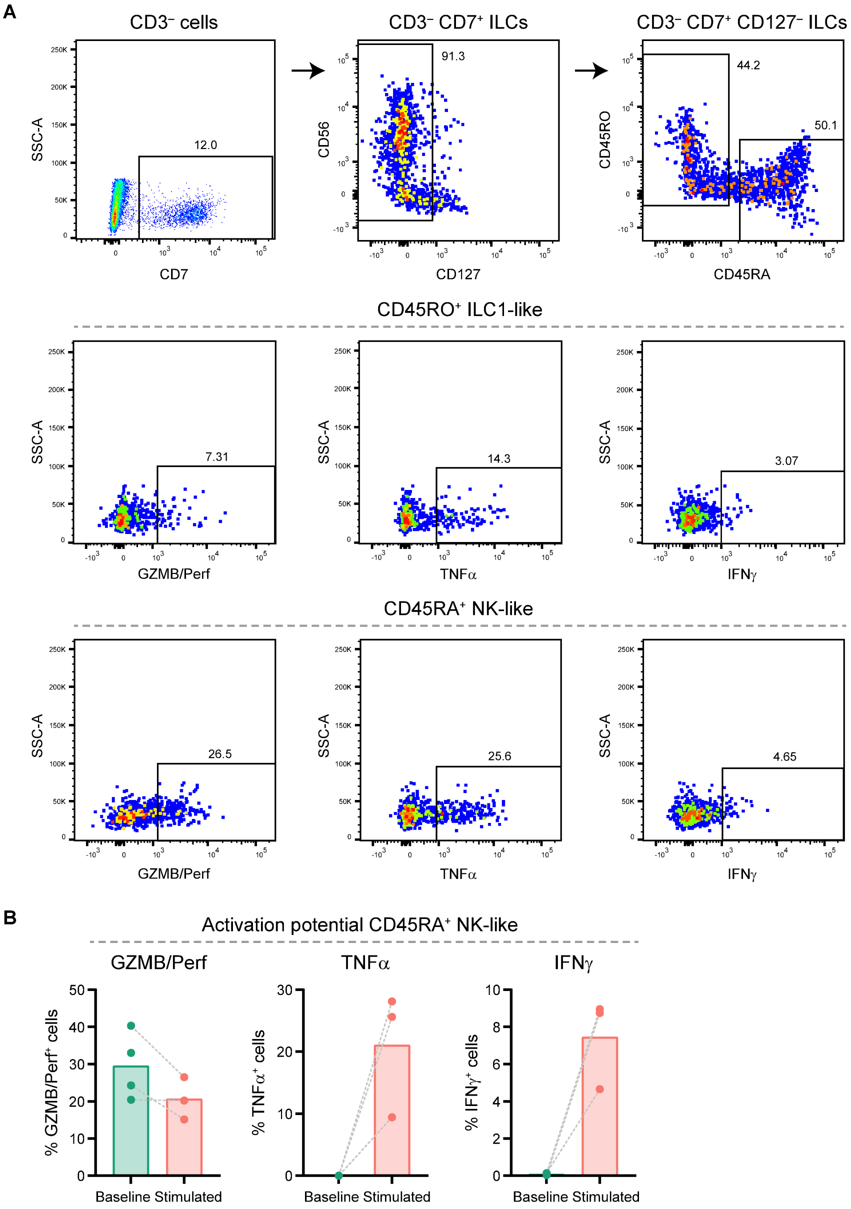

Supplemental figure 7. Gating strategy for the detection of cytotoxic molecules and cytokines by ILCs. A. Flow cytometry gating strategy to detect granzyme B/perforin, TNFα, and IFNγ in CD45RO⁺ ILC1-like cells and CD45RA⁺ NK-like cells of a representative pancreatic tumor sample (upon stimulation with PMA/ionomycin) showing sequential gates with percentages, starting with the CD3⁻ gate from supplemental figure 6. **B.** Cytokine production by CD45RA⁺ NK-like cells from PDAC tissues *ex vivo* (N=4) and upon stimulation with PMA/ionomycin (N=3) measured by flow cytometry. Bars indicate the mean and lines indicate matched samples.

Supplemental figure 8

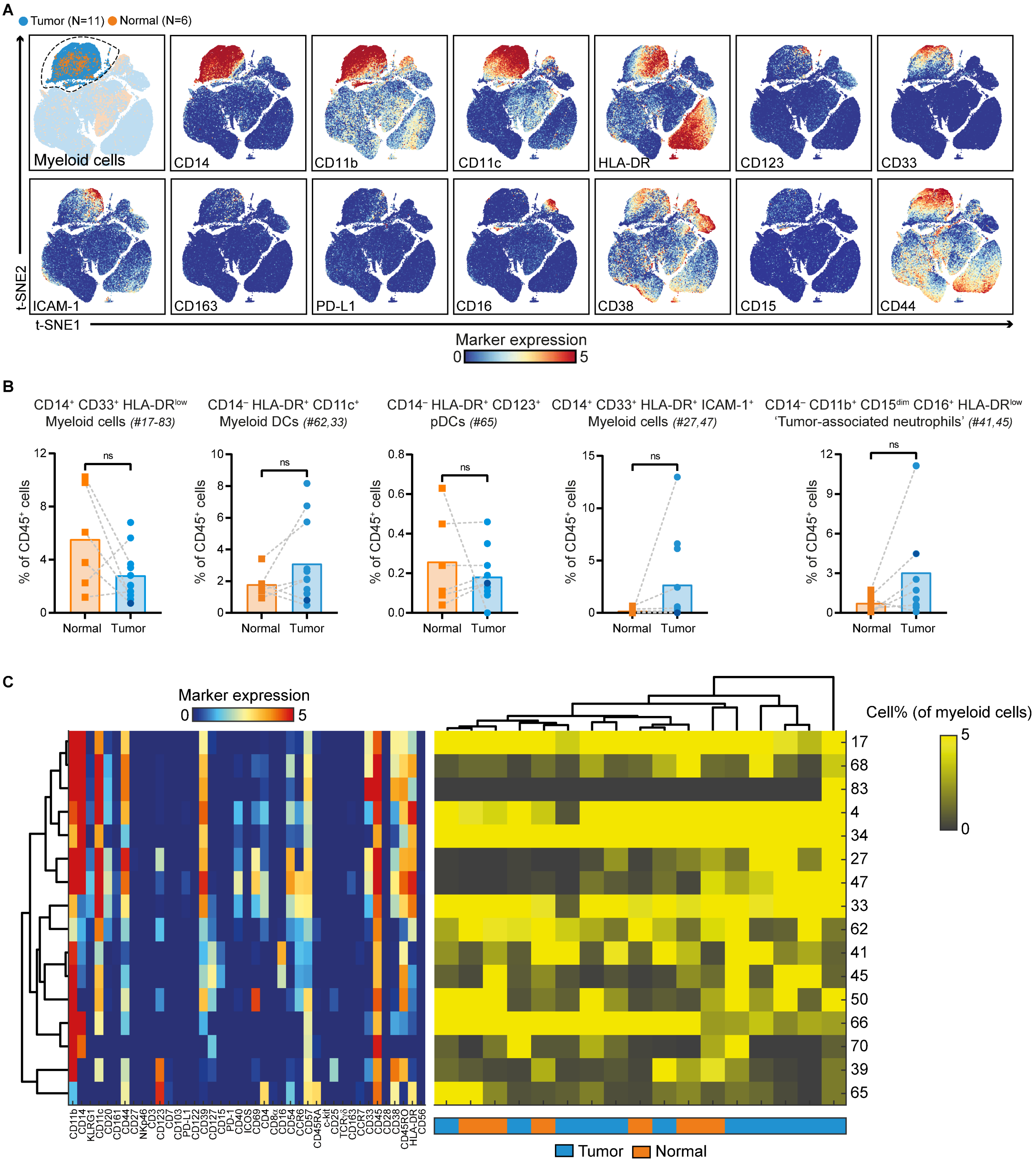

Supplemental figure 8. Myeloid cell phenotypes identified in PDAC and non-malignant pancreatic tissues. A. t-SNE embedding from figure 1A highlighting the myeloid cells, colored by tissue type (first plot) and relative expression of indicated myeloid cell markers (remainder). **B.** Frequencies of selected myeloid cell clusters in PDAC tissues (N=11) as compared to non-malignant pancreatic tissues (N=6) shown as percentage of total CD45⁺ cells. Cluster IDs correspond to the ones in (**C**). Each dot represents an individual sample (dark blue color represents patient ISPIC20). Bars indicate the mean and lines indicate matched samples. Data from 11 independent experiments with mass cytometry. **C.** A heatmap showing median marker expression values (left) and frequencies of all identified myeloid cell clusters among PDAC and non-malignant pancreatic tissues (right). Hierarchical clustering was performed on cluster frequencies using Spearman's rank correlation.

Supplemental figure 9

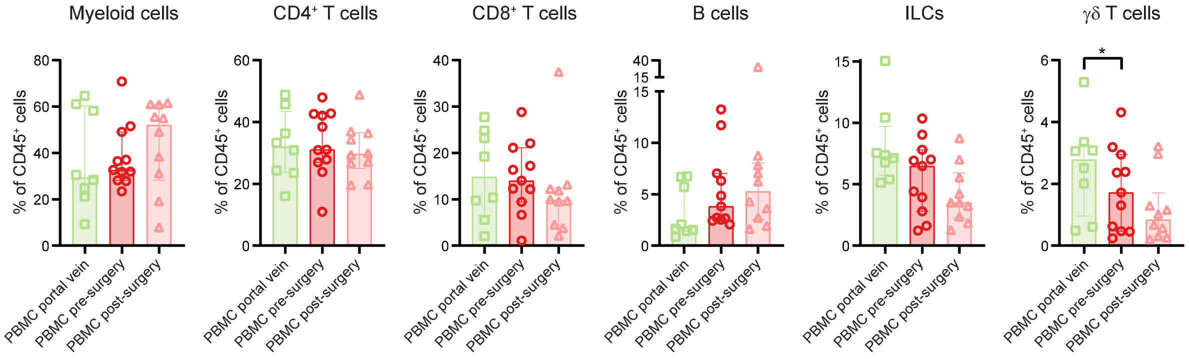

Supplemental figure 9. Frequency of the major immune lineages in portal vein and peripheral blood from patients with PDAC. Frequencies of the major immune lineages in portal vein blood (N=8) and peripheral blood before (N=11) as well as after (N=10) surgery from 11 patients with PDAC shown as percentage of total CD45$^+$ cells. Each dot represents an individual sample. Bars indicate the median with IQR. Data from 11 independent experiments with mass cytometry. *P<0.05 by Wilcoxon test.

Supplemental figure 10

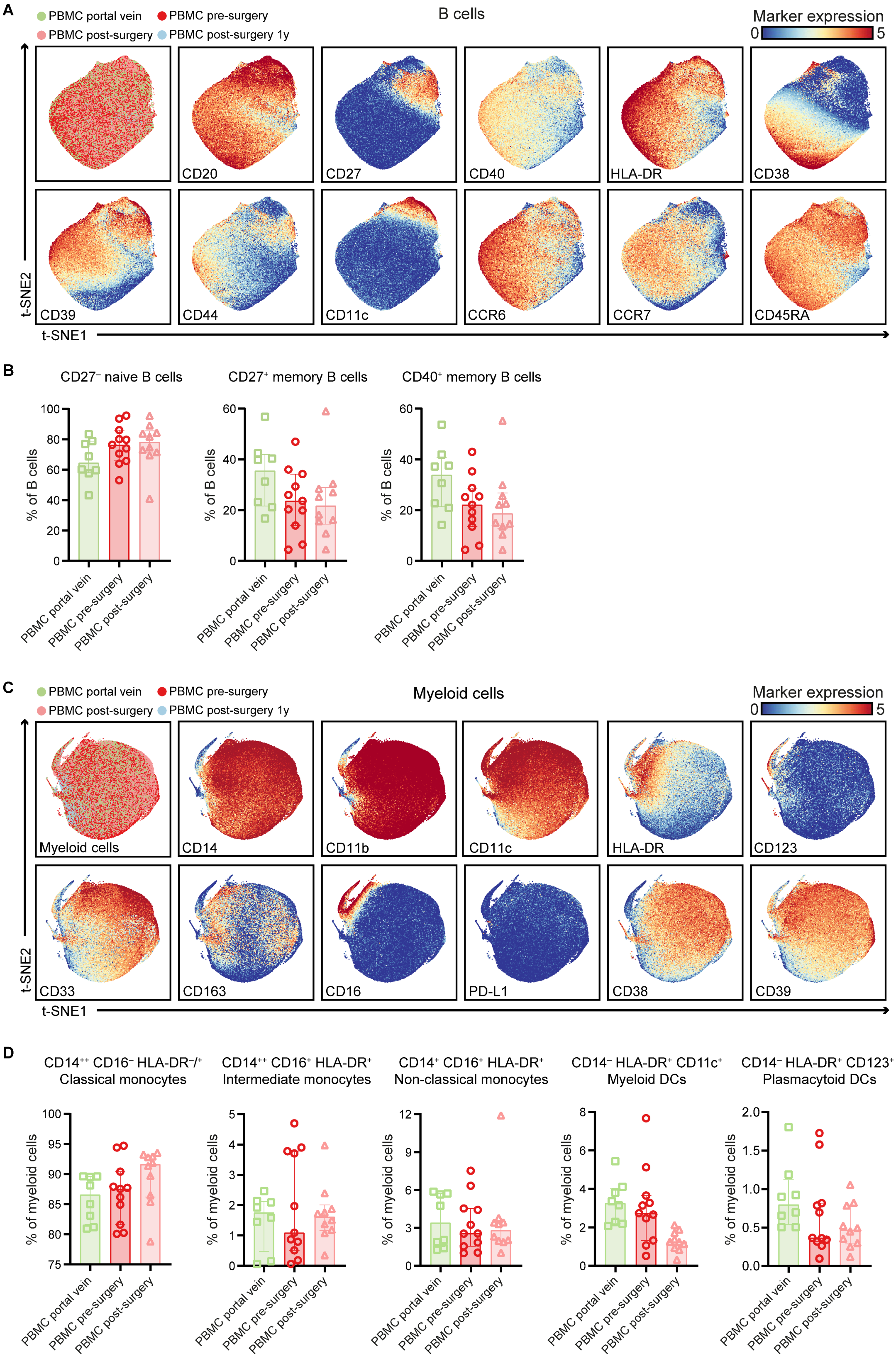

Supplemental figure 10. Overview of B cell and myeloid cell phenotypes in portal vein and peripheral blood from patients with PDAC. A. t-SNE embedding showing 363,140 B cells isolated from portal vein blood (N=8), peripheral blood before surgery (N=11), peripheral blood directly after surgery (N=10) as well as one year after surgery (N=1) from 11 patients with PDAC clustered based on the expression of 41 immune cell markers by single-cell mass cytometry. The cells are colored by tissue type (first plot) and relative expression of indicated B cell markers (remainder). **B.** Frequencies of general B cell populations in the different blood samples as percentage of total B cells. **C.** t-SNE embedding showing 499,980 myeloid cells isolated from the same samples as in (**A**). The cells are colored by tissue type (first plot) and relative expression of indicated myeloid cell markers (remainder). **D.** Frequencies of general myeloid cell populations in the different blood samples as percentage of total myeloid cells. **B,D.** Each dot represents an individual sample. Bars indicate the median with IQR. Data from 11 independent experiments with mass cytometry.

Supplemental figure 11

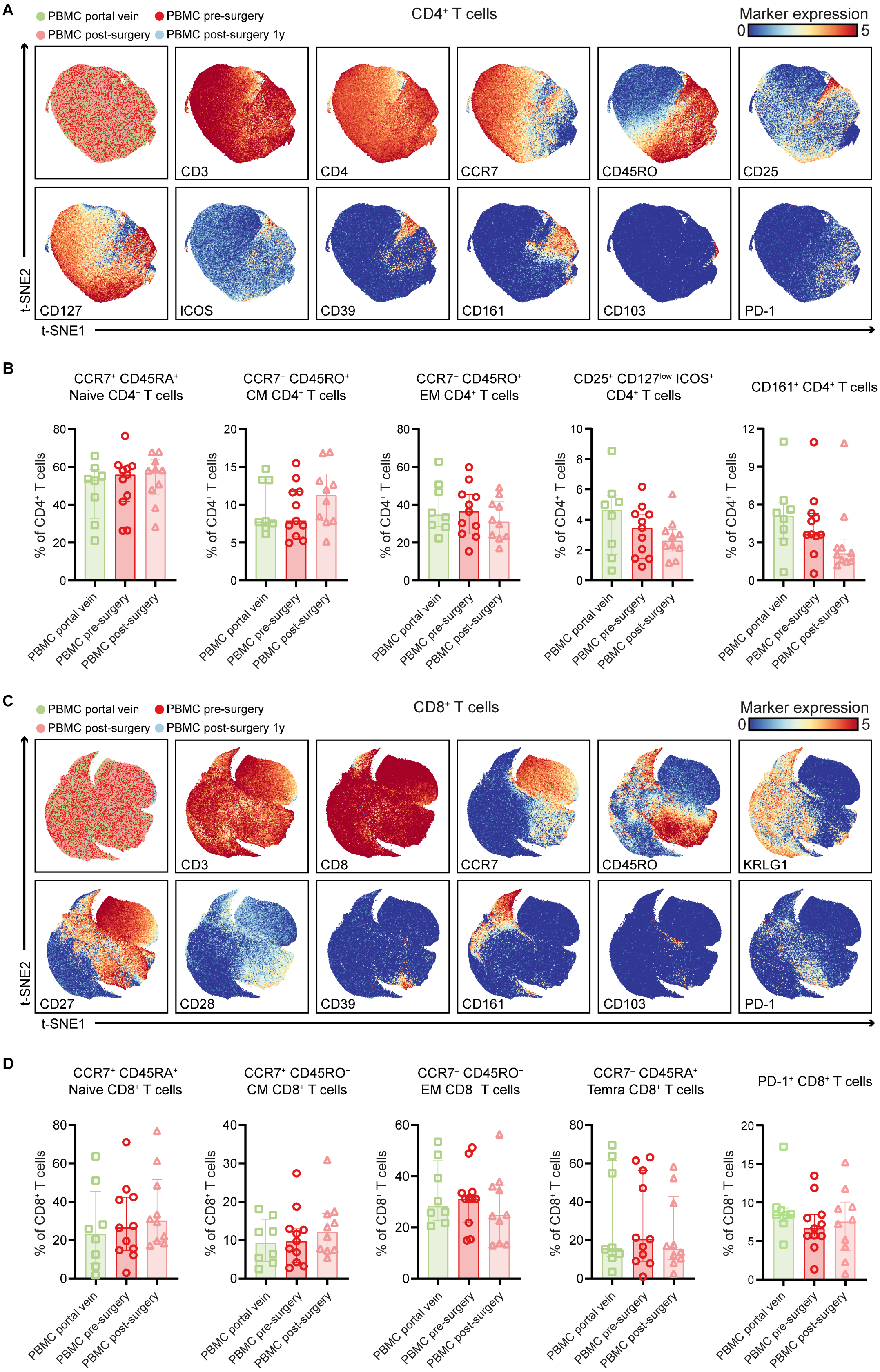

Supplemental figure 11. Overview of $CD4^+$ and $CD8^+$ T cell phenotypes in portal vein and peripheral blood from patients with PDAC. A. t-SNE embedding showing 499,980 $CD4^+$ T cells isolated from portal vein blood (N=8), peripheral blood before surgery (N=11), peripheral blood directly after surgery (N=10) as well as one year after surgery (N=1) from 11 patients with PDAC clustered based on the expression of 41 immune cell markers by single-cell mass cytometry. The cells are colored by tissue type (first plot) and relative expression of indicated $CD4^+$ T cell markers (remainder). **B.** Frequencies of selected $CD4^+$ T cell clusters in different blood samples as percentage of total $CD4^+$ T cells. **C.** t-SNE embedding showing 445,521 $CD8^+$ T cells isolated from the same samples as in (**A**). The cells are colored by tissue type (first plot) and relative expression of indicated $CD8^+$ T cell markers (remainder). **D.** Frequencies of selected $CD8^+$ T cell clusters in different blood samples as percentage of total $CD8^+$ T cells. **B,D.** Each dot represents an individual sample. Bars indicate the median with IQR. Data from 11 independent experiments with mass cytometry.

Supplementary table 1. Antibodies used for mass cytometry experiments.

Mass cytometry					
**Antibodies**	**Metal**	**Clone**	**Source**	**Identifier**	**Dilution**
Anti-human CD45	^{89}Y	HI30	Fluidigm	Cat# 3089003B	1/100
Anti-human CD14	$^{112/114}$Cd	Tük4	ThermoFisher Scientific	Cat# Q10064	1/1000
Anti-human CD15	^{115}In	W6D3	BioLegend	Cat# 323035	1/50
Anti-human CD196/CCR6	^{141}Pr	G034E3	Fluidigm	Cat# 3141003A	1/50
Anti-human CD40	^{142}Nd	5C3	Fluidigm	Cat# 3142010B	1/50
Anti-human CD278/ICOS	^{143}Nd	C398.4A	Fluidigm	Cat# 3143025B	1/50
Anti-human CD69	^{144}Nd	FN50	Fluidigm	Cat# 3144018B	1/100
Anti-human CD4	^{145}Nd	RPA-T4	Fluidigm	Cat# 3145001B	1/200
Anti-human CD8α	^{146}Nd	RPA-T8	Fluidigm	Cat# 3146001B	1/200
Anti-human c-kit	^{147}Sm	104D2	BioLegend	Cat# 313223	1/50
Anti-human CD16	^{148}Nd	3G8	Fluidigm	Cat# 3148004B	1/100
Anti-human CD25	^{149}Sm	2A3	Fluidigm	Cat# 3149010B	1/100
Anti-human CD54/ICAM-1	^{150}Nd	HCD54	BioLegend	Cat# 322702	1/200
Anti-human CD123/IL-3Rα	^{151}Eu	6H6	Fluidigm	Cat# 3151001B	1/100
Anti-human TCRγδ	^{152}Sm	11F2	Fluidigm	Cat# 3152008B	1/50
Anti-human CD7	^{153}Eu	CD7-6B7	Fluidigm	Cat# 3153014B	1/100
Anti-human CD163	^{154}Sm	GHI/61	Fluidigm	Cat# 3154007B	1/50
Anti-human CD103	^{155}Gd	Ber-ACT8	Sony	Cat# 2351010	1/100
Anti-human CD274/PD-L1	^{156}Gd	29E.2A3	Fluidigm	Cat# 3156026B	1/50
Anti-human CD122/IL-2Rβ	^{158}Gd	TU27	BioLegend	Cat# 339015	1/100
Anti-human CD197/CCR7	^{159}Tb	G043H7	Fluidigm	Cat# 3159003A	1/100
Anti-human CD39	^{160}Gd	A1	Fluidigm	Cat# 3160004B	1/200
Anti-human KLRG1	^{161}Dy	REA261	Miltenyi Biotec	Cat# 120-014-229	1/50
Anti-human CD11c	^{162}Dy	Bu15	Fluidigm	Cat# 3162005B	1/200
Anti-human CD20	^{163}Dy	2H7	BioLegend	Cat# 302343	1/300
Anti-human CD161	^{164}Dy	HP-3G10	Fluidigm	Cat# 3164009B	1/100
Anti-human CD127/IL-7Rα	^{165}Ho	AO19D5	Fluidigm	Cat# 3165008B	1/200
Anti-human CD44	^{166}Er	BJ18	Fluidigm	Cat# 3166001B	1/200
Anti-human CD27	^{167}Er	O323	Fluidigm	Cat# 3167002B	1/150
Anti-human CD335/NKp46	^{168}Er	9E2	Sony	Cat# 2259510	1/50
Anti-human CD33	^{169}Tm	WM53	Fluidigm	Cat# 3169010B	1/200
Anti-human CD3	^{170}Er	UCHT1	Fluidigm	Cat# 3170001B	1/200
Anti-human CD28	^{171}Yb	CD28.2	BioLegend	Cat# 302937	1/100
Anti-human CD38	^{172}Yb	HIT2	Fluidigm	Cat# 3172007B	1/200
Anti-human CD45RO	^{173}Yb	UCHL1	BioLegend	Cat# 304239	1/50
Anti-human HLA-DR	^{174}Yb	L243	Fluidigm	Cat# 3174001B	1/400
Anti-human CD279/PD-1	^{175}Lu	EH12.2H7	Fluidigm	Cat# 3175008B	1/100
Anti-human CD56	^{176}Yb	NCAM16.2	Fluidigm	Cat# 3176008B	1/100
Anti-human CD57	^{194}Pt	HCD57	BioLegend	Cat# 322325	1/200
Anti-human CD45RA	^{198}Pt	HI100	BioLegend	Cat# 304143	1/200
Anti-human CD11b	^{209}Bi	ICRF44	Fluidigm	Cat# 3209003B	1/100
